# Point prevalence assessments of five respiratory viruses in adults ≥ 50 years of age in Germany by multi-pathogen rapid antigen testing on June 1, 2024, and December 15, 2024

**DOI:** 10.1007/s15010-026-02917-5

**Published:** 2026-08-25

**Authors:** Miranda Anggitakirana, Julia A. Nacov, Jon Salmanton-García, Julia Jakobs, Sarah Grimm, Janina Leckler, Katharina Köbe, Louise M. Cremer, Pauline Wipfler, Jannik Stemler, Heinz-Josef Schmitt, Ullrich Bethe, Oliver A. Cornely

**Affiliations:** 1 Faculty of Medicine and University Hospital Cologne, Institute of Translational Research, Cologne Excellence Cluster on Cellular Stress Responses in Aging-Associated Diseases (CECAD), University of Cologne, Herderstr. 52, 50931 Cologne, Germany; 2 Faculty of Medicine, Department I of Internal Medicine, Excellence Center for Medical Mycology (ECMM), University of Cologne, and University Hospital Cologne, Cologne, Germany; 3 German Centre for Infection Research (DZIF), Partner Site Bonn-Cologne, Cologne, Germany; 4 Faculty of Medicine, University of Cologne, University Hospital Cologne, Clinical Trials Centre Cologne (ZKS Köln), Cologne, Germany

**Keywords:** Rapid antigen test, MAK5, Viral respiratory tract infections, AdV, SARS-CoV-2, Influenza

## Abstract

**Purpose:**

Acute respiratory infections (ARI) impose a significant burden on healthcare systems and society. This study aimed to investigate the circulation of respiratory viruses in community-dwelling older adults in Germany.

**Methods:**

Participants aged ≥ 50 years in Germany performed multi-pathogen rapid antigen self-tests (MAK5) detecting adenovirus (AdV), severe acute respiratory syndrome coronavirus 2 (SARS‑CoV‑2), influenza A and B, and respiratory syncytial virus (RSV) on June 1 and December 15, 2024. Participants reported test results, ARI symptoms and duration, medical consultations, work absenteeism, comorbidities, vaccination history, and prior infections.

**Results:**

2,654 participants enrolled in the point prevalence assessment on June 1, 2024 and 2,385 on December 15, 2024. Most tests were negative (*n* = 4,807; 93.9%), with an overall detection rate of 5.7%. In June, 153 pathogens were detected, compared to 138 in December. AdV was the most frequently detected pathogen in both June (59/153; 38.6%) and December (44/138; 31.9%), followed by SARS-CoV-2 and influenza A. Among participants with a positive test, at least one ARI symptom was reported by 47.7% in June and 58.7% in December. SARS-CoV-2 was associated with the highest clinical burden, resulting in healthcare utilization in 10.0% and 19.4% of cases and work absenteeism in 10.0% and 13.9% of cases in June and December, respectively.

**Conclusion:**

Point prevalence testing revealed substantial AdV circulation, underrepresented in routine surveillance. Its frequent co-detection with other viruses suggests a role as a co-infecting agent. Community-based screening can help identify asymptomatic reservoirs and underreported viral circulation, linking viral detection to clinical and economic impact.

**Supplementary Information:**

The online version contains supplementary material available at https://doi.org/10.1007/s15010-026-02917-5.

## Introduction

Acute respiratory infections (ARI) are highly prevalent and impose a substantial burden on healthcare systems and society. Despite long-standing institutional surveillance systems in multiple countries, comprehensive community-level monitoring remains limited [1]. Between 1990 and 2021, the global age-standardized incidence rates for upper respiratory infections ranged from 166,771 to 175,481 cases per 100,000 population [2]. Viral pathogens, including adenovirus (AdV), influenza viruses, respiratory syncytial virus (RSV), and severe acute respiratory syndrome coronavirus 2 (SARS-CoV-2), represent the most common etiological viral agents [3]. While most ARI remain asymptomatic or result in mild clinical courses, their high prevalence, economic impact and potential for severe outcomes highlight the need for systematic tracking of viral circulation patterns and dynamics to guide targeted prevention strategies, including vaccination [4, 5].

Older adults and individuals with chronic underlying and/or immunocompromising conditions are particularly vulnerable, with immunosenescence and inflammaging contributing to increased risk of severe disease, high rates of medical consultations, hospitalizations, and overall morbidity [6, 7]. However, even in these high-risk populations, routine diagnostic testing is not universally implemented, as it remains confined to medical institutions due to legal, technical and operational constraints [8, 9]. Consequently, conventional surveillance systems may underestimate pathogen prevalences, while failing to capture co-circulation patterns or associations with pre-existing conditions [9, 10]. Previous community-based studies assessing point prevalences have already shown higher viral detections than reported in institutional data [11].

Since the COVID-19 pandemic, rapid antigen tests (RAT) have become widely available, well accepted, and cost-efficient tools for detecting viral pathogens, offering rapid and reliable results. Although less sensitive than nucleic acid amplification tests (NAATs), RATs have undergone technological improvements, achieving high specificity and increasingly reliable sensitivity, and are now available in multiplex formats, allowing the simultaneous detection of multiple endemic respiratory pathogens from a single respiratory sample [12].

Using multi-pathogen antigen test kits (MAK5), this study aimed to assess point prevalences of five endemic viruses in adults ≥ 50 years in Germany, and to evaluate underlying health conditions.

## Methods

### Participant recruitment

Participant recruitment was conducted through V-Reg, a European online database of adult volunteers willing to participate in clinical studies and other research initiatives [13]. By March 2024, the registry included over 36,000 adult volunteers. A total of 10,187 volunteers meeting eligibility criteria (residence in Germany, age ≥ 50 years, access to a computer, smartphone, or tablet for electronic consent, and ability to upload photos) were invited to participate via email.

### Study procedures

Of those invited, 3,162 volunteers provided electronic informed consent and completed an online survey detailing their demographic characteristics and baseline health information. During the study period, participant-reported baseline information, including vaccination history and underlying medical conditions, was updated upon receipt of new or revised information submitted via email.

Participants received MAK5 test kits for self-testing by mail between April and May 2024 (Supplementary Fig. 1), along with detailed instructions and symptom diaries (Supplementary Figs. 2 and ). The MAK5 test (clinical performance summarized in Supplementary Table 1) is an immunochromatographic lateral flow device (LFD) designed to detect AdV, influenza A and B viruses, RSV, and SARS-CoV-2 from a single nasal specimen (BioTeke Corporation (Wuxi) Co., Ltd., Wuxi, Jiangsu, China) [14]. Participants were instructed to self-administer the MAK5 test on June 1, 2024, and December 15, 2024, regardless of symptoms, to obtain point prevalence estimates at two predefined time points in different periods of the year. The testing schedule was communicated at the outset of the study, and participants received multiple reminders to ensure adherence and maximize compliance.

### Data collection

Data were collected using a web-based eCRF developed and managed via www.clinicalsurveys.net (EFS Fall 2022, TIVIAN, Cologne, Germany). The eCRF recorded participants’ interpretation of the test result and, for those experiencing symptoms, detailed information on symptom profiles, ARI episode duration, number of medical consultations, frequency and duration of hospitalizations and ICU stays, hours on mechanical ventilation, and days of sick leave, if applicable. Participants were also asked to submit a photograph of the test strip to allow verification of test results.

### Statistical analysis

The required sample size was calculated a priori for the German population aged ≥ 50 years (*n* = 37,668,559). Calculations were based on an RSV prevalence of 7.1%, derived from our findings from the MAK-5 pilot study point prevalence assessment on June 1, 2023 [15]. Building on a margin of error or absolute precision of ± 1%, as recommended for large-scale studies, and at 95% confidence (corresponding to 6.0% to 8.0%) the required sample size was 2,501. Accounting for an anticipated 26.4% drop-out rate in our cohort based on the pilot study, the final enrolment target was set at 3,162 participants.

The data were analysed using SPSS version 27.0 (IBM Corp., Chicago, IL, USA). Categorical variables were presented as frequencies and percentages, while continuous variables were summarized as median values with interquartile ranges (IQR) and absolute ranges.

## Results

**Fig. 1:**
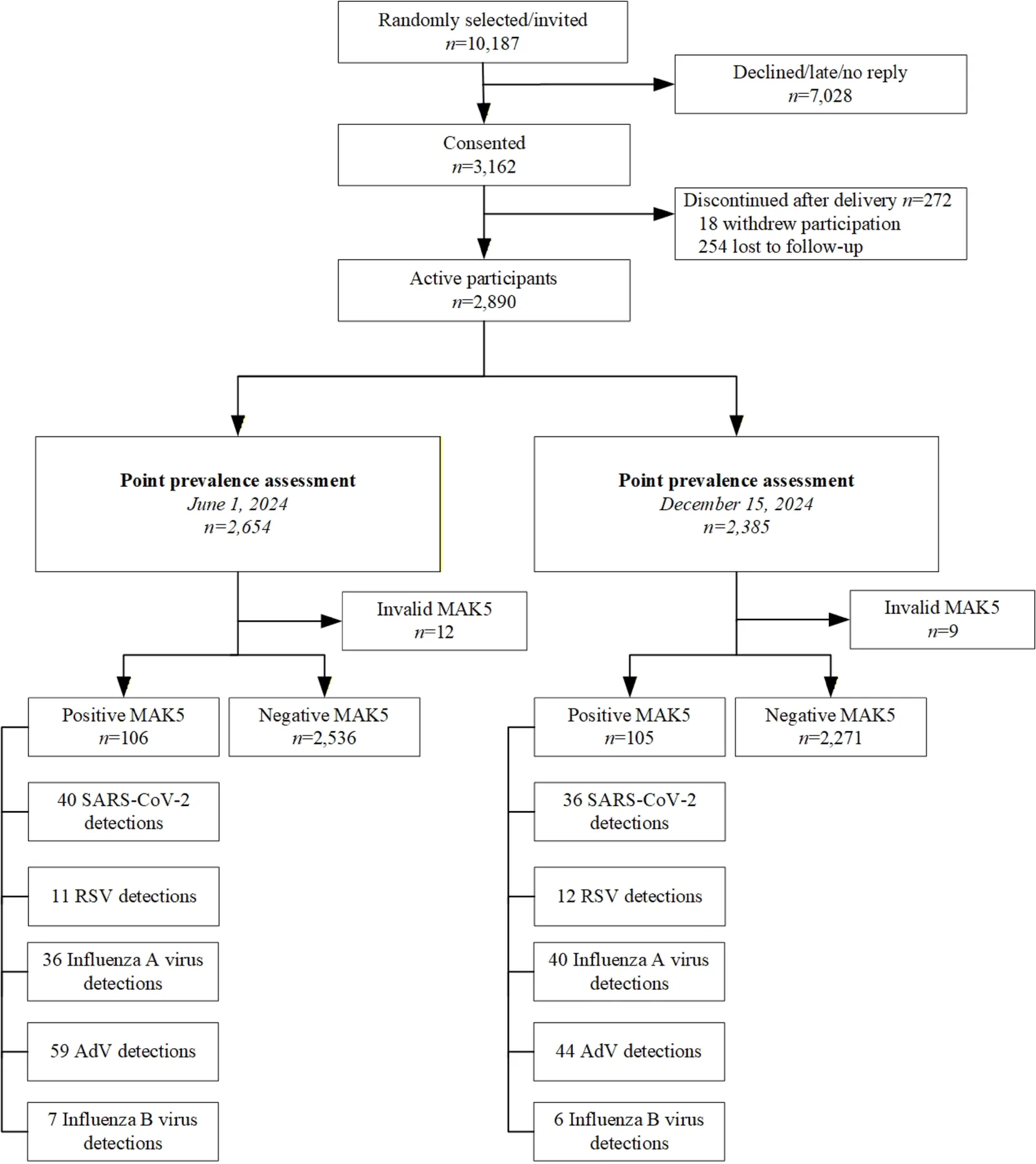
Participant flow diagram

This study enrolled 3,162 individuals aged ≥ 50 years in Germany who provided baseline characteristics and informed consent via eCRF between April and May 2024 and consequently received MAK5 test kits via mail. Of these, 2,890 (91.4%) were categorized as active participants, as they collaborated in at least one of the two point prevalence assessments (Fig. 1).

### Cohort characteristics

The cohort comprised 1,675 (58%) women, 1,214 (42.0%) men, and 1 non-binary^1^ person. The participants’ median age was 59 years (IQR: 55–65 years; range 50–91 years).

The most common comorbidities were hypertension (*n* = 785; 27.2%), chronic pulmonary disease (*n* = 317; 11.0%), overweight ≥ 20 kg/m² (*n* = 330; 11.4%), and diabetes mellitus (*n* = 121; 4.2%).

The largest proportion of participants resided in the federal state of North Rhine-Westphalia (*n* = 1,348; 46.6%), followed by individuals from Baden-Württemberg (*n* = 271; 9.4%), Bavaria (*n* = 241; 8.3%) and Hesse (*n* = 236; 8.2%).

Further details on geographical distribution, other underlying conditions, previous viral respiratory infections, and relevant vaccination records of all active participants as well as stratified by participation in the June and December point prevalence assessments are provided in Table 1.

**Table 1:** Cohort characteristics

	Participants on June 1, 2024 *N* [%]		Participants on December 15, 2024 *N* [%]		Active Participants *N* [%]	
TOTAL	2654	[91.8]	2385	[91.8]	2890	[100.0]
**Gender**						
Female	1552	[58.5]	1374	[57.6]	1675	[58.0]
Male	1101	[41.5]	1010	[42.3]	1214	[42.0]
Non-binary*	1	[0.0]	1	[0.0]	1	[0.0]
**Age (years)**						
Median (IQR) [min-max]	**59**	**(55–65) [50–91]**	**60**	**(55–66) [50–91]**	**59**	**(55–65) [50–91]**
**Age groups**						
50 to 60 years	1378	[51.9]	1181	[49.5]	1490	[51.6]
60 to 70 years	941	[35.5]	888	[37.2]	1029	[35.6]
70 to 80 years	302	[11.4]	290	[12.2]	330	[11.4]
80 years and above	33	[1.2]	26	[1.1]	41	[1.4]
**Place of residence**						
Baden-Württemberg	236	[8.9]	231	[9.7]	271	[9.4]
Bavaria	226	[8.5]	197	[8.3]	241	[8.3]
Berlin	123	[4.6]	107	[4.5]	128	[4.4]
Brandenburg	49	[1.8]	51	[2.1]	57	[2.0]
Bremen	19	[0.7]	18	[0.8]	21	[0.7]
Hamburg	46	[1.7]	43	[1.8]	52	[1.8]
Hesse	220	[8.3]	189	[7.9]	236	[8.2]
Lower Saxony	167	[6.3]	149	[6.2]	178	[6.2]
Mecklenburg-Western Pomerania	19	[0.7]	18	[0.8]	21	[0.7]
North Rhine-Westphalia	1246	[46.9]	1119	[46.9]	1348	[46.6]
Rhineland-Palatinate	116	[4.4]	109	[4.6]	130	[4.5]
Saarland	15	[0.6]	14	[0.6]	15	[0.5]
Saxony	49	[1.8]	47	[2.0]	57	[2.0]
Saxony-Anhalt	30	[1.1]	26	[1.1]	32	[1.1]
Schleswig-Holstein	64	[2.4]	45	[1.9]	69	[2.4]
Thuringia	29	[1.1]	22	[0.9]	34	[1.2]
**Pre-existing conditions**						
Hypertension	714	[26.9]	630	[26.4]	785	[27.2]
Coronary heart disease	97	[3.7]	85	[3.6]	103	[3.6]
Heart failure	38	[1.4]	39	[1.6]	43	[1.5]
Asthma/COPD/Chronic bronchitis/emphysema	292	[11.0]	254	[10.6]	317	[11.0]
Chronic hepatitis B or C	0	[0.0]	0	[0.0]	0	[0.0]
Chronic non-infectious liver disease including cirrhosis	17	[0.6]	14	[0.6]	17	[0.6]
Chronic kidney disease	35	[1.3]	32	[1.3]	40	[1.4]
≥ 20 kg overweight	309	[11.6]	249	[10.4]	330	[11.4]
Diabetes mellitus	114	[4.3]	94	[3.9]	121	[4.2]
HIV	22	[0.8]	22	[0.9]	25	[0.9]
Active cancer (current therapy or within last 2 yrs)	59	[2.2]	53	[2.2]	62	[2.1]
Epilepsy	13	[0.5]	14	[0.6]	13	[0.4]
Chronic GIT disease	29	[1.1]	22	[0.9]	30	[1.0]
Severe musculoskeletal condition	61	[2.3]	42	[1.8]	65	[2.2]
Psychiatric condition	137	[5.2]	110	[4.6]	143	[4.9]
History of stroke	21	[0.8]	22	[0.9]	25	[0.9]
Other	787	[29.7]	714	[29.9]	865	[29.9]
**Previously confirmed infections at any timepoint**						
Influenza	243	[9.2]	206	[8.6]	263	[9.1]
COVID-19	2061	[77.7]	1857	[77.9]	2249	[77.8]
RSV infection	2	[0.1]	2	[0.1]	2	[0.1]
AdV infection	11	[0.4]	8	[0.3]	11	[0.4]
**Seasonal influenza vaccination**	2097	[79.0]	1915	[80.3]	2283	[79.0]
Last influenza vaccination received in 2024	755	[28.4]	716	[30.0]	828	[28.7]
Last influenza vaccination received in 2023	1074	[40.5]	965	[40.5]	1164	[40.3]
Last influenza vaccination received in 2022	155	[5.8]	132	[5.5]	170	[5.9]
Last influenza vaccination received in 2021	61	[2.3]	54	[2.3]	61	[2.1]
Last influenza vaccination received in 2020	27	[1.0]	26	[1.1]	31	[1.1]
Last influenza vaccination received in 2019	25	[0.9]	22	[0.9]	29	[1.0]
**COVID-19 vaccination**	2606	[98.2]	2349	[98.5]	2840	[98.3]
Number of total COVID-19 vaccinations Median (IQR) [min-max]	4	(3–5) [0–10]	4	(3–5) [0–10]	4	(3–5) [0–10]
Last COVID-19 vaccination received in 2024	396	[14.9]	398	[16.7]	441	[15.3]
Last COVID-19 vaccination received in 2023	789	[29.7]	707	[29.6]	846	[29.3]
Last COVID-19 vaccination received in 2022	789	[29.7]	683	[28.6]	859	[29.7]
Last COVID-19 vaccination received in 2021	602	[22.7]	543	[22.8]	663	[22.9]
Last COVID-19 vaccination received in 2020	5	[0.2]	3	[0.1]	5	[0.2]
Last COVID-19 vaccination received = BioNTech	2115	[79.7]	1924	[80.7]	2308	[79.9]
Last COVID-19 vaccination received = Moderna	420	[15.8]	365	[15.3]	455	[15.7]
Last COVID-19 vaccination received = Other	71	[2.7]	60	[2.5]	77	[2.7]
**RSV vaccination**	42	[1.6]	40	[1.7]	49	[1.7]
Last received RSV vaccination in 2024	27	[1.0]	26	[1.1]	31	[1.1]
Last received RSV vaccination in 2023	15	[0.6]	14	[0.6]	18	[0.6]

*AdV* adenovirus, *COPD* chronic obstructive pulmonary disease, *COVID-19* coronavirus disease 2019, *GIT* gastrointestinal, *HIV* human immunodeficiency virus, *INF* infection, *RSV* respiratory syncytial virus, *SARS-CoV-2* severe acute respiratory syndrome coronavirus 2, *SD* standard deviation

*Corresponds to legal term “divers” in German language

### Cumulative prevalences

Across both point prevalence assessments, a total of 5,119 MAK5 test results were submitted. Most tests were negative (*n* = 4,807 out of 5,119; 93.9%), with an overall pathogen detection rate of 5.7% (*n* = 291). AdV was most frequently detected (*n* = 103; 2.0%), followed by SARS-CoV-2 and influenza A virus (*n* = 76; 1.5%), RSV (*n* = 23; 0.4%), and influenza B virus (*n* = 13; 0.3%) (Supplementary Table 2).

**Fig. 2:**
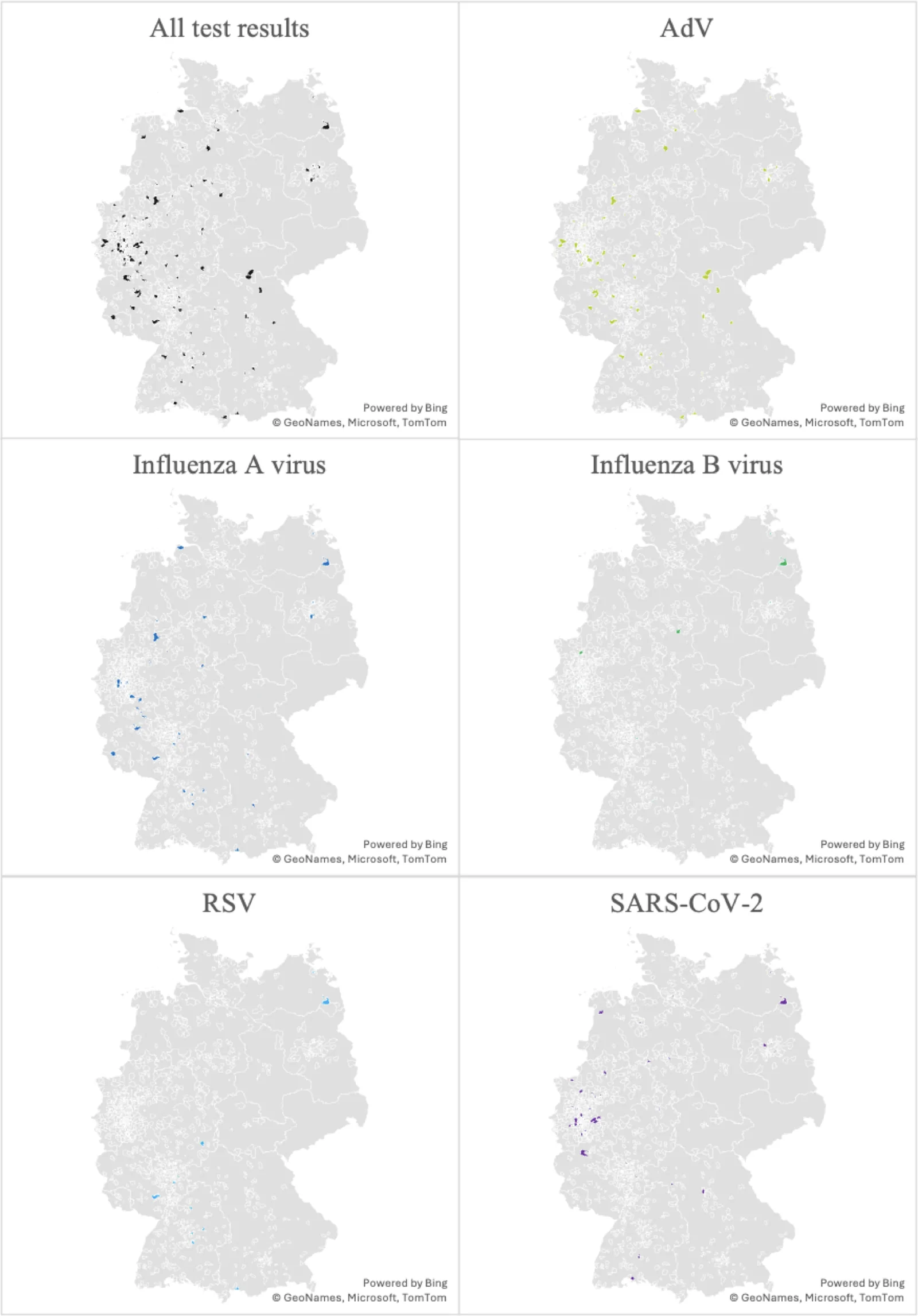
Geographical distribution of MAK5 test results on point prevalence assessments from June 1, 2024, and December 15, 2024. *AdV* adenovirus, *RSV* respiratory syncytial virus, *SARS-CoV-2* severe acute respiratory syndrome coronavirus 2, *MAK5* 5 in 1 multiple respiratory multipathogen antigen test kit

**Fig. 3:**
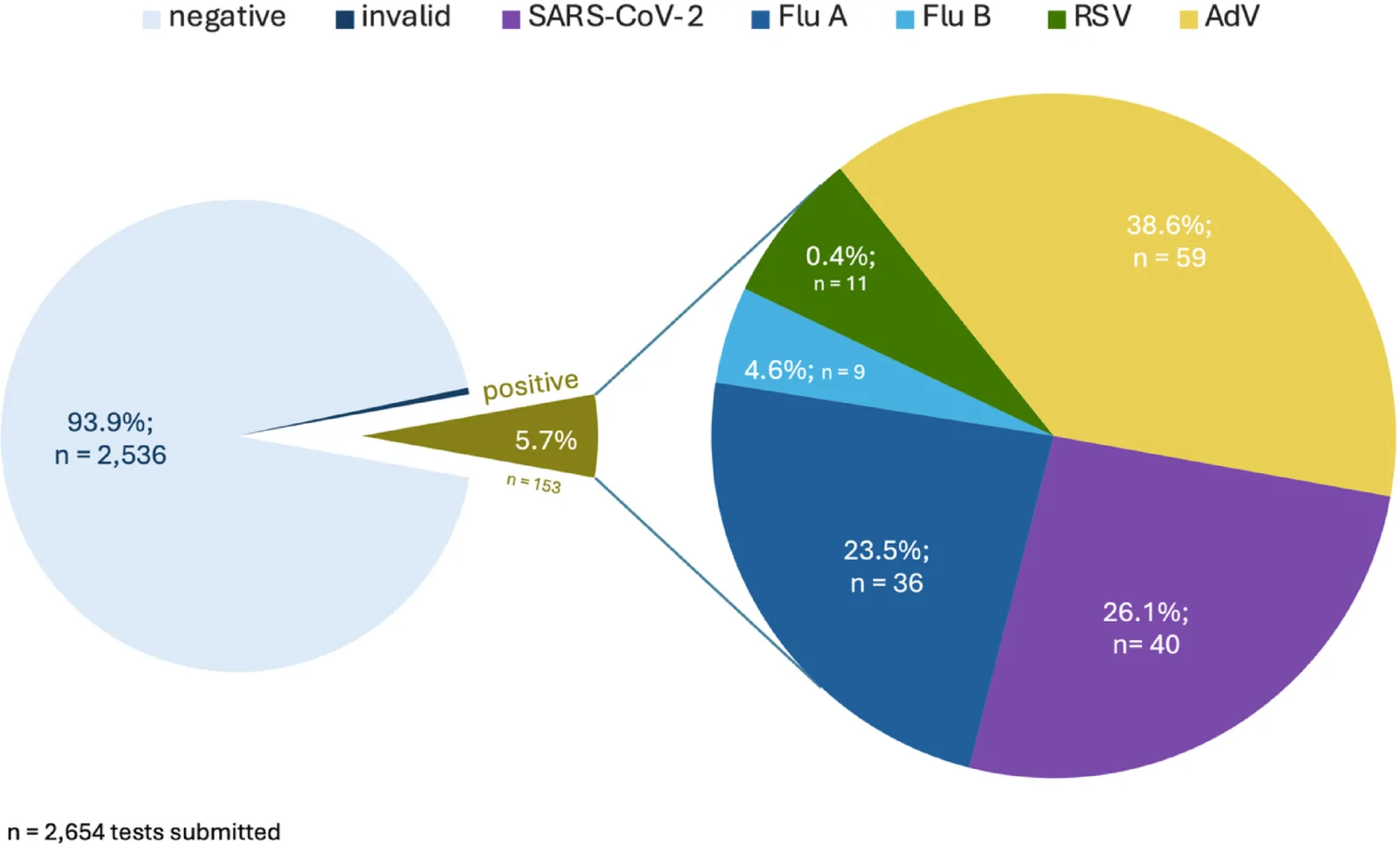
Distribution of MAK5 test results on June 1, 2024. *AdV* adenovirus, *RSV* respiratory syncytial virus, *SARS-CoV-2* severe acute respiratory syndrome coronavirus

**Fig. 4:**
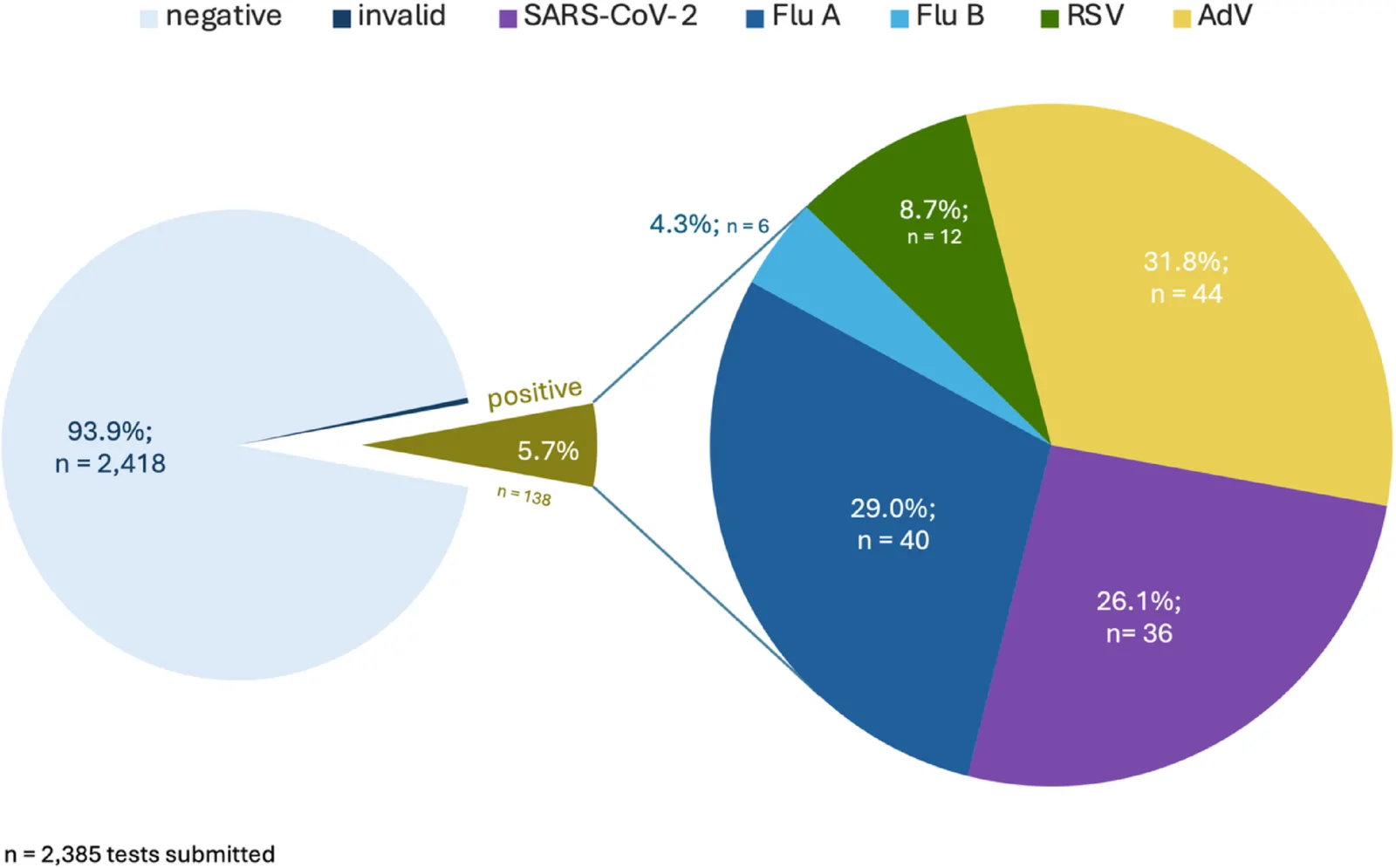
Distribution of MAK5 test results on December 15, 2024. *AdV* adenovirus, *RSV* respiratory syncytial virus, *SARS-CoV-2* severe acute respiratory syndrome coronavirus, *MAK5* 5 in 1 multiple respiratory multipathogen antigen test kit

Co-detections were reported in 49 tests, while 162 tests detected a single pathogen. AdV was most frequently detected alongside other viruses (*n* = 44 out of 49; 89.8%), primarily with influenza A virus (*n* = 42 out of 44; 95.5%) (Supplementary Table 3). Single pathogen detections were led by SARS-CoV-2 (*n* = 64 out of 162; 39.5%) and AdV (*n* = 58 out of 162; 35.8%) (Supplementary Table 4). Geographical distribution of cumulative prevalences is illustrated in Fig. 2, while diagrams of pathogen distributions can be found in Figs. 3 and 4 for the June and December assessment respectively.

### Point prevalence assessment on June 1, 2024

Of the 2,890 active participants, 2,654 (91.8%) performed and submitted a test result on June 1, 2024. Among these, 2,536 out of 2,654 (95.6%) tests were negative and 106 (4.0%) were positive, resulting in 153 (5.8% out of 2,654) viral detections, including 29 (27.4% out of 106) co-detections.

AdV was the most frequently detected virus (*n* = 59 out of 153; 38.6%; point prevalence: 2.2%), followed by influenza A virus (*n* = 36 out of 153; 23.5%; point prevalence: 1.4%) and SARS-CoV-2 (*n* = 40 out of 153; 26.1%; point prevalence: 1.5%). RSV and Influenza B virus detections were rare, with eleven (7.2% out of 153; point prevalence: 0.4%) and seven (4.6% out of 153; point prevalence: 0.3%) detections respectively, while twelve tests were invalid (0.5% out of 2,654).

Detailed symptom profiles, healthcare utilization and economic impact are detailed in Table 2. Detailed cohort characteristics including demographic data, preexisting conditions and vaccination records are presented in Supplementary Table 5 while geographical distribution of the participants is provided in Supplementary Tables 6 and Supplementary Fig. 4.

**Table 2:** Symptom profiles, healthcare utilization, and economic impact among participants by test result from point prevalence assessment on June 1, 2024

	Participants with invalid test *N* [%]	Participants with negative test *N* [%]	Participants positive for SARS-CoV-2 *N* [%]	Participants positive for Influenza A virus *N* [%]	Participants positive for Influenza B virus *N* [%]	Participants positive for RSV *N* [%]	Participants positive for AdV *N* [%]	TOTAL *N* [%]
TOTAL	12 [0.4]	2536 [93.9]	40 [1.5]	36 [1.3]	7 [0.3]	11 [0.4]	59 [2.2]	2701 [100.0]
**Symptoms**								
Cough	0 [0.0]	27 [1.1]	0 [0.0]	1 [2.8]	0 [0.0]	0 [0.0]	1 [1.7]	29 [1.1]
Pharyngitis	0 [0.0]	95 [3.7]	9 [22.5]	3 [8.3]	0 [0.0]	2 [18.2]	7 [11.9]	116 [4.3]
Rhinitis	0 [0.0]	190 [7.5]	19 [47.5]	5 [13.9]	0 [0.0]	2 [18.2]	17 [28.8]	233 [8.6]
Dyspnea	0 [0.0]	62 [2.4]	3 [7.5]	4 [11.1]	0 [0.0]	1 [9.1]	9 [15.3]	79 [2.9]
Conjunctivitis	0 [0.0]	60 [2.4]	6 [15.0]	1 [2.8]	1 [14.3]	1 [9.1]	5 [8.5]	74 [2.7]
Anosmia/Ageusia	0 [0.0]	31 [1.2]	6 [15.0]	0 [0.0]	0 [0.0]	0 [0.0]	1 [1.7]	38 [1.4]
Fever	0 [0.0]	20 [0.8]	4 [10.0]	0 [0.0]	0 [0.0]	0 [0.0]	0 [0.0]	24 [0.9]
Asthenia	0 [0.0]	124 [4.9]	20 [50.0]	7 [19.4]	1 [14.3]	3 [27.3]	13 [22.0]	168 [6.2]
Hoarseness	0 [0.0]	74 [2.9]	5 [12.5]	1 [2.8]	0 [0.0]	0 [0.0]	5 [8.5]	85 [3.2]
Chills	0 [0.0]	18 [0.7]	6 [15.0]	0 [0.0]	0 [0.0]	0 [0.0]	1 [1.7]	25 [0.9]
GIT symptoms	1 [8.3]	38 [1.5]	3 [7.5]	0 [0.0]	0 [0.0]	0 [0.0]	1 [1.7]	43 [1.6]
Ear involvement	0 [0.0]	32 [1.3]	5 [12.5]	3 [8.3]	0 [0.0]	1 [9.1]	5 [8.5]	46 [1.7]
Vertigo	0 [0.0]	34 [1.3]	6 [15.0]	2 [5.6]	0 [0.0]	2 [18.2]	3 [5.1]	47 [1.7]
Sneezing	0 [0.0]	90 [3.5]	13 [32.5]	3 [8.3]	0 [0.0]	1 [9.1]	10 [16.9]	117 [4.3]
Lymphadenopathy	0 [0.0]	24 [0.9]	3 [7.5]	0 [0.0]	0 [0.0]	0 [0.0]	1 [1.7]	28 [1.0]
Sputum production	0 [0.0]	106 [4.2]	8 [20.0]	1 [2.8]	0 [0.0]	0 [0.0]	6 [10.2]	121 [4.5]
Subfebrile temperature	0 [0.0]	20 [0.8]	7 [17.5]	0 [0.0]	0 [0.0]	0 [0.0]	1 [1.7]	28 [1.0]
Tonsilitis	0 [0.0]	16 [0.6]	2 [5.0]	0 [0.0]	0 [0.0]	0 [0.0]	1 [1.7]	19 [0.7]
Sinus pressure	0 [0.0]	58 [2.3]	7 [17.5]	2 [5.6]	0 [0.0]	0 [0.0]	7 [11.9]	74 [2.7]
Arthralgia/Myalgia	0 [0.0]	55 [2.2]	7 [17.5]	1 [2.8]	0 [0.0]	0 [0.0]	3 [5.1]	66 [2.4]
Dry eye/ocular pain	0 [0.0]	68 [2.7]	5 [12.5]	0 [0.0]	0 [0.0]	0 [0.0]	2 [3.4]	75 [2.8]
Cephalgia	0 [0.0]	45 [1.8]	3 [7.5]	4 [11.1]	0 [0.0]	2 [18.2]	5 [8.5]	59 [2.2]
Other	0 [0.0]	6 [0.2]	1 [2.5]	1 [2.7]	0 [0.0]	1 [9.1]	1 [1.7]	10 [0.4]
**Medical care/economic burden**								
Number of medical consultations	0 [0.0]	34 [1.3]	4 [10.0]	1 [2.8]	0 [0.0]	1 [9.1]	2 [3.4]	42 [1.6]
Days on sick leave	0 [0.0]	24 [0.9]	4 [10.0]	1 [2.8]	0 [0.0]	1 [9.1]	2 [3.4]	32 [1.2]

*AdV* adenovirus, *ARI* acute respiratory infection, *GIT* gastrointestinal, *ICU* intensive care unit, *IQR* interquartile range, *RSV* respiratory syncytial virus, *SARS-CoV-2* severe acute respiratory syndrome coronavirus 2

### Point prevalence assessment on December 15, 2024

2,385 (82.5%) out of 2,890 active participants performed the MAK5 test on December 15, 2024, which resulted in 2,271 (95.2% out of 2,385) negative and 105 (4.4% out of 2,385) positive test results. Among the positive results, there were 20 (19.0% out of 105) cases of co-detections, resulting in a total of 138 (5.8% out of 2,385) viral detections.

AdV was once again the most frequently detected virus (*n* = 44 out of 138; 31.9%; point prevalence: 1.8%), this time followed by influenza A virus (*n* = 40 out of 138; 29.0%; point prevalence: 1.7%), SARS-CoV-2 (*n* = 36 out of 138; 26.1%; point prevalence: 1.5%), and RSV (*n* = 12 out of 138; 8.7%; point prevalence: 0.5%). Influenza B virus was detected in six cases (4.3% out of 138; point prevalence: 0.3%) only, and nine tests (0.4% out of 2,385) were invalid.

Detailed symptom profiles, healthcare utilization and economic impact of the cohort are detailed in Table 3. Detailed cohort characteristics including demographic data, pre-existing medical conditions and vaccination records are presented in Supplementary Table 7, while geographical distribution of the participants is provided in Supplementary Tables 8 and Supplementary Fig. 5.

**Table 3:** Symptom profiles, healthcare utilization, and economic impact among participants by test result from point prevalence assessment on December 15, 2024

	Participants with invalid test *N* [%]	Participants with negative test *N* [%]	Participants positive for SARS-CoV-2 *N* [%]	Participants positive for Influenza A virus *N* [%]	Participants positive for Influenza B virus *N* [%]	Participants positive for RSV *N* [%]	Participants positive for AdV *N* [%]	TOTAL *N* [%]
TOTAL	9 [0.4]	2271 [93.9]	36 [1.5]	40 [1.7]	6 [0.2]	12 [0.5]	44 [1.8]	2418 [100.0]
**Symptoms**								
Cough	1 [11.1]	138 [6.1]	13 [36.1]	5 [12.5]	0 [0.0]	2 [16.7]	7 [15.9]	166 [6.9]
Pharyngitis	2 [22.2]	325 [14.3]	25 [69.4]	12 [30.0]	0 [0.0]	4 [33.3]	14 [31.8]	382 [15.8]
Rhinitis	0 [0.0]	68 [3.0]	9 [25.0]	4 [10.0]	1 [16.7]	1 [8.3]	6 [13.6]	89 [3.7]
Dyspnea	0 [0.0]	55 [2.4]	8 [22.2]	3 [7.5]	0 [0.0]	0 [0.0]	2 [4.5]	68 [2.8]
Conjunctivitis	0 [0.0]	37 [1.6]	9 [25.0]	4 [10.0]	0 [0.0]	0 [0.0]	1 [2.3]	51 [2.1]
Anosmia/Ageusia	0 [0.0]	20 [0.9]	5 [13.9]	4 [10.0]	0 [0.0]	1 [8.3]	2 [4.5]	32 [1.3]
Fever	2 [22.2]	159 [7.0]	18 [50.0]	8 [20.0]	0 [0.0]	1 [8.3]	10 [22.7]	198 [8.2]
Asthenia	0 [0.0]	93 [4.1]	12 [33.3]	8 [20.0]	1 [16.7]	1 [8.3]	6 [13.6]	121 [5.0]
Hoarseness	0 [0.0]	23 [1.0]	8 [22.2]	4 [10.0]	0 [0.0]	1 [8.3]	4 [9.1]	40 [1.7]
Chills	0 [0.0]	50 [2.2]	9 [25.0]	4 [10.0]	1 [16.7]	1 [8.3]	1 [2.3]	66 [2.7]
GIT symptoms	0 [0.0]	36 [1.6]	7 [19.4]	3 [7.5]	1 [16.7]	1 [8.3]	2 [4.5]	50 [2.1]
Ear involvement	0 [0.0]	36 [1.6]	7 [19.4]	4 [10.0]	0 [0.0]	1 [8.3]	1 [2.3]	49 [2.0]
Vertigo	1 [11.1]	118 [5.2]	16 [44.4]	7 [17.5]	0 [0.0]	2 [16.7]	5 [11.4]	149 [6.2]
Sneezing	0 [0.0]	27 [1.2]	3 [8.3]	3 [7.5]	0 [0.0]	1 [8.3]	1 [2.3]	35 [1.4]
Lymphadenopathy	1 [11.1]	142 [6.3]	11 [30.6]	8 [20.0]	1 [16.7]	2 [16.7]	4 [9.1]	169 [7.0]
Sputum production	0 [0.0]	24 [1.1]	6 [16.7]	3 [7.5]	0 [0.0]	1 [8.3]	2 [4.5]	36 [1.5]
Subfebrile temperature	0 [0.0]	14 [0.6]	4 [11.1]	1 [2.5]	0 [0.0]	0 [0.0]	0 [0.0]	19 [0.8]
Tonsilitis	0 [0.0]	71 [3.1]	12 [33.3]	5 [12.5]	1 [16.7]	2 [16.7]	7 [15.9]	98 [4.1]
Sinus pressure	0 [0.0]	75 [3.3]	12 [33.3]	5 [12.5]	0 [0.0]	1 [8.3]	2 [4.5]	95 [3.9]
Arthralgia/Myalgia	1 [11.1]	65 [2.9]	8 [22.2]	3 [7.5]	0 [0.0]	2 [16.7]	4 [9.1]	83 [3.4]
Dry eye/ocular pain	1 [11.1]	40 [1.8]	2 [5.6]	1 [2.5]	0 [0.0]	0 [0.0]	3 [6.8]	47 [1.9]
Cephalgia	0 [0.0]	5 [0.2]	0 [0.0]	1 [2.5]	0 [0.0]	0 [0.0]	2 [4.5]	8 [0.3]
Other	0	8	2	0	0	0	0	10 [0.4]
**Medical care/economic burden**								
Number of medical consultations	0 [0.0]	54 [2.4]	7 [19.4]	2 [5.0]	1 [16.7]	1 [8.3]	4 [9.1]	69 [2.9]
Days on sick leave	0 [0.0]	61 [2.7]	5 [13.9]	2 [5.0]	1 [16.7]	1 [8.3]	6 [13.6]	76 [3.2]

*AdV* adenovirus, *ARI* acute respiratory infection, *GIT* gastrointestinal, *ICU* intensive care unit, *IQR* interquartile range, *RSV* respiratory syncytial virus, *SARS-CoV-2* severe acute respiratory syndrome coronavirus 2

### Comparison of the June and December point prevalence assessments and symptom profiles

Influenza A virus was detected in 36 of 2,654 participants (1.3%) during the June point prevalence assessment and in 40 of 2,385 participants (1.7%) during the December assessment. Conversely, AdV detections decreased from 59 of 2,654 (2.2%) in June to 44 of 2,385 (1.8%) in December (Supplementary Table 2).

Overall, the proportion of symptomatic participants was low at both point prevalence assessments, with fewer symptoms reported in June compared with December. In total, 435 out of 2,654 participants (16.4%) in June and 535 out of 2,385 (22.4%) in December reported at least one ARI symptom, corresponding to 2,219 (83.6%) and 1,850 (77.6%) asymptomatic individuals, respectively. Among symptomatic participants, 73 out of 435 (16.8%) had a positive MAK5 test result in June, and 81 out of 535 (15.1%) recorded at least one pathogen detection in December.

SARS-CoV-2 was associated with the highest proportion of symptomatic detections, affecting 26 out of 40 participants (65.0%) in June and 30 out of 36 participants (83.3%) in December. Influenza A virus exhibited relatively low symptom prevalence, with 12 out of 36 participants (33.3%) symptomatic in June and 19 out of 40 participants (47.5%) in December. For influenza B, symptoms were reported in 1 out of 7 participants (14.3%) in June and 3 out of 6 participants (50.0%) in December. RSV infections were symptomatic in 5 out of 11 participants (45.5%) in June and 6 out of 12 participants (50.0%) in December. In contrast, AdV was most frequently detected in asymptomatic individuals, representing 29 out of 59 participants (49.2%) in June and 23 out of 44 participants (52.3%) in December.

The distribution of reported symptoms differed between the two point prevalence assessments. In June, rhinitis (233 out of 2,701; 8.6%), asthenia (168 out of 2,701; 6.2%), and pharyngitis (116 out of 2,701; 4.3%) were most common. In December, pharyngitis (382 out of 2,418; 15.8%), fever (198 out of 2,418; 8.2%), cough (166 out of 2,418; 6.9%), and rhinitis (89 out of 2,418; 3.7%) predominated (Tables 2 and 3).

### Clinical and economic impact

In June, 42 (1.6% out of 2,654) participants sought medical consultations for ARI, and 32 (1.2% out of 2,654) reported sick leave. The highest percentage in these two categories were recorded by participants with SARS-CoV-2 detection, with 4 of 40 (10.0%) sought medical care and 4 (10.0%) received sick leave. For RSV, 1 of 11 (9.1%) had a medical consultation and took sick leave, while for AdV 2 of 59 (3.4%) participants sought medical consultations, and 2 (3.4%) reported sick leave. These rates were lowest among those with influenza A, with 1 of 36 (2.8%) had a medical consultation and 1 (2.8%) took sick leave. No cases among influenza B virus–positive participants sought medical care or absence from work in June.

In December, a higher number of healthcare utilization and cases of sickness-related absence from work can be observed. Medical consultations were reported in 69 (2.9% out of 2,385), and sick leave in 76 (3.2% out of 2,385) cases. SARS-CoV-2-positive participants once again demonstrated the highest rate of healthcare utilization, with 7 (19.4% out of 36) sought medical consultation and 5 (13.9% out of 36) could not work. AdV was associated with 4 of 44 (9.1%) medical consultations and 6 of 44 (13.6%) work absence. For influenza A, 2 of 40 (5.0%) sought medical consultation and 2 (5.0%) reported sick leave, while influenza B had 1 of 6 (16.7%) for both outcomes. Among RSV-positives, 1 of 12 (8.3%) sought medical attention and 1 (8.3%) reported sick leave.

Further clinical outcomes are summarized in Tables 2 and 3 for June and December assessments respectively, with results being categorized by test results.

## Discussion

The present study provides a community-based assessment of five endemic respiratory viruses in older adults outside of a medical setting, based on two point prevalence assessments conducted on June 1 and December 15, 2024. The same cohort was invited to complete both assessments; however, participation was voluntary, and not all individuals contributed data at both time points. Of 2,890 active participants, 2,149 (74.4%) of participants participated in both assessments (Supplementary Fig. 6).

Across the two assessments, AdV emerged as the most frequently detected pathogen, and was the predominant co-detected virus. Overall, 4.2% of participants aged ≥ 50 years tested positive for at least one MAK5 pathogen, reflecting otherwise moderate viral shedding, in line with low point prevalence reported in other community-based cohorts [16, 17]. Notably, 47.1% of all detections occurred in asymptomatic participants, indicating that asymptomatic viral detection may represent an underrecognized component of respiratory virus circulation at the community level.

This high prevalence of AdV contrasts with data from institutional surveillance in Germany, particularly from the Robert Koch Institute (RKI) ARI Sentinel, where only one AdV detection was reported across all age groups in calendar week 22 and 50 [18, 19]. This discrepancy is likely explained by the structure of sentinel surveillance, which relies on reports from outpatient practices, hospitals, and diagnostic laboratories. Therefore, highly symptomatic cases are captured as testing is usually restricted to hospitalized patients or to decide whether a patient should be hospitalized and receive further diagnostics and/or treatment. In addition, AdV frequently presents asymptomatically, as shown above. This is compatible with the pathobiology of species C AdV, which can establish persistent or latent infections in lymphoid tissues, with viral DNA detectable long after primary infection and the capacity for reactivation in the absence of clinical symptoms [20]. Older adults further often present atypical respiratory infections with non-specific symptoms such as decreased mobility, frailty, or delirium, which, combined with low healthcare-seeking behaviour, further reduces detection in conventional surveillance [21, 22]. Inclusion of asymptomatic infections in our cohort highlights the value of decentralized, community-based monitoring to better characterize respiratory virus circulation beyond clinically symptomatic infections [18, 19, 23].

Although AdV outbreaks are generally observed in children or military, respiratory infections caused by AdV remain clinically relevant to the general population due to its risks of complicated clinical progression especially in immunocompromised individuals [24, 25]. While co-infections with AdV have been previously described in paediatric populations, evidence in older adults remains scarce [24]. On a molecular level, AdV-encoded proteins such as E1B 55-kDa may facilitate co-infections by modulating host immune responses mediating transcriptional repression of IFN-inducible genes, though further research is needed to clarify these interactions [26].

Comparison of the two point prevalence assessments demonstrated a higher prevalence of virus detection, a greater proportion of participants reporting respiratory symptoms and increased healthcare utilization in December compared with June. Influenza A virus and RSV were detected more frequently in December, consistent with their well-established seasonal epidemiology. SARS-CoV-2 prevalence remained relatively stable across the two assessments, suggesting endemic circulation, while AdV was the only virus detected with reduced frequency in December, consistent with its less pronounced seasonal pattern [27, 28].

These patterns must be interpreted in the context of persistently elevated sickness-related absence from work in Germany in the post-pandemic period [29, 30]. Official statistics report an average of 14.8 sickness-related workdays per employee in 2024, representing a substantial increase compared with pre-pandemic years and underscoring the sustained burden of illness-related absenteeism on the workforce, with respiratory infections serving as a major driver [29, 31, 32]. Collectively, these trends have fuelled ongoing public and policy debate regarding the economic and societal consequences of sick leave and the appropriate balance between infection control, presenteeism, and workforce stability in Germany [33, 34].

In healthcare settings, pandemic-related absence from work was substantial and driven not only by confirmed infections, but also by precautionary quarantine and individual risk perception, contributing to staff shortages and operational strain [32, 35, 36]. These observations highlight how lack of knowledge of infection status can amplify absenteeism beyond medically necessary sick leave.

In this context, accessible and timely viral testing may represent a complementary strategy to support informed decision-making regarding work attendance, particularly for respiratory infections with heterogeneous or mild clinical presentations. By enabling earlier identification of viral infections at the community level, decentralized testing approaches could help reduce unnecessary absence from work while simultaneously limiting workplace transmission, thereby addressing both public health and economic considerations during periods of heightened respiratory virus circulation.

The findings of our study need to be interpreted in the context of certain limitations. First, although MAK5 tests are user-friendly and provide high specificity, they remain less sensitive than NAAT and do not detect other common respiratory pathogens such as rhinovirus, other human coronaviruses, and human metapneumovirus [3, 14, 37]. In addition, differences in analytical sensitivity among the target viruses may have led to underestimation of the prevalence of individual pathogens.

Second, self-reported symptoms may introduce reporting bias and potential misclassification.

Third, the manufacturer’s reported sensitivity and specificity data were derived from a mixed adult and paediatric population and therefore might not be fully representative for older populations [38].

Fourth, the sample size calculation was calculated to achieve high precision for the expected RSV prevalence based on our previous MAK5 point prevalence assessment on June 1, 2023, due to its emerging public health relevance following the introduction of RSV vaccination in older adults [15]. Consequently, while the cohort provided valuable descriptive data on the circulation of multiple respiratory viruses, prevalence estimates for less frequently detected pathogens are inherently less precise.

Fifth, because this study was limited to two predefined point prevalence assessments, the findings represent snapshots of pathogen circulation at two discrete time points and cannot fully characterize seasonal dynamics. To complement these findings, longitudinal data from the same cohort, capturing symptom-based testing over a one-year period, have been reported separately to address temporal transmission dynamics of the MAK5 pathogens [39].

In conclusion, this study provides a community-based assessment of five endemic respiratory viruses at two predefined time points in older adults, capturing both symptomatic and asymptomatic test detections. Its main contribution lies in integrating pathogen-specific data with demographic characteristics, underlying medical conditions, vaccination history, and clinical as well as economic outcomes, providing a broader understanding of disease burden complementing conventional sentinel surveillance. The systematic inclusion of asymptomatic infections enables the detection of otherwise missed viral circulation, generating complementary data to routine surveillance systems and informing public health interventions and targeted vaccination strategies in this vulnerable population.

## Supplementary Information

Below is the link to the electronic supplementary material.

Supplementary Material 1

## Data Availability

Data available on request from the corresponding author.

## Footnotes

1 Corresponds to the German term *divers*.

## References

[1] Gupta S, Gupta T, Gupta N. Global respiratory virus surveillance: strengths, gaps, and way forward. Int J Infect Dis. 2022;121:184–9. 10.1016/j.ijid.2022.05.032. PMC9107382 35584744

[2] Chen C, You Y, Du Y, Zhou W, Jiang D, Cao K, et al. Global epidemiological trends in the incidence and deaths of acute respiratory infections from 1990 to 2021. Heliyon. 1990;10(16):e35841. 10.1016/j.heliyon.2024.e35841. PMC11367038 39224281

[3] Manno M, Pavia G, Gigliotti S, Pantanella M, Barreca GS, Peronace C, et al. Respiratory virus prevalence across pre-, during-, and post-SARS-CoV-2 pandemic periods. Viruses. 2025. 10.3390/v17081040. PMC12390457 40872755

[4] Branche A, Ramesh M, Francis B. A narrative review of key risk factors for severe illness following SARS-CoV-2, influenza virus, and respiratory syncytial virus infection. Infect Dis Therapy. 2025;14(1):39–61. 10.1007/s40121-024-01081-3. PMC11724830 39739198

[5] Mok DZL, Chan CYY, Ooi EE, Chan KR. The effects of aging on host resistance and disease tolerance to SARS-CoV-2 infection. FEBS J. 2021;288(17):5055–70. 10.1111/febs.15613. PMC8518758 33124149

[6] Boe DM, Boule LA, Kovacs EJ. Innate immune responses in the ageing lung. Clin Exp Immunol. 2017;187(1):16–25. 10.1111/cei.12881. PMC5167032 27711979

[7] Chen Y, Klein SL, Garibaldi BT, Li H, Wu C, Osevala NM, et al. Aging in COVID-19: vulnerability, immunity and intervention. Ageing Res Rev. 2021;65:101205. 10.1016/j.arr.2020.101205. PMC7604159 33137510

[8] Klompas M, Imrey PB, Yu PC, Rhee C, Deshpande A, Haessler S, et al. Respiratory viral testing and antibacterial treatment in patients hospitalized with community-acquired pneumonia. Infect Control Hosp Epidemiol. 2021;42(7):817–25. 10.1017/ice.2020.1312. PMC8665830 33256870

[9] Branche AR, Falsey AR. Viral diagnostics: only half the battle. J Infect Dis. 2017;216(8):923–5. 10.1093/infdis/jix289. PMC5853519 28633488

[10] Ljubin-Sternak S, Meštrović T, Lukšić I, Mijač M, Vraneš J. Seasonal coronaviruses and other neglected respiratory viruses: a global perspective and a local snapshot. Front Public Health. 2021. 10.3389/fpubh.2021.691163. PMC8287126 34291031

[11] Stemler J, Salmanton-García J, Weise B, Többen C, Joisten C, Fleig J, et al. A pilot surveillance report of SARS-CoV-2 rapid antigen test results among volunteers in Germany, 1st week of July 2022. Infection. 2022;51(2):465–9. 10.1007/s15010-022-01931-7. PMC9590393 36279033

[12] Boukli N, Flamand C, Chea KL, Heng L, Keo S, Sour K, et al. One assay to test them all: Multiplex assays for expansion of respiratory virus surveillance. Front Med. 2023. 10.3389/fmed.2023.1161268. PMC10165998 37168265

[13] Salmanton-García J, Stewart FA, Heringer S, Koniordou M, Álvarez-Barco E, Argyropoulos CD, et al. VACCELERATE volunteer registry: a European study participant database to facilitate clinical trial enrolment. Vaccine. 2022;40(31):4090–7. 10.1016/j.vaccine.2022.05.022. PMC9159788 35659449

[14] BioTeke. 5 In 1 Multiple Respiratory Multipathogen Antigen Test Kit [SARS-CoV-2, Influenza A/B, RSV, ADV; anterior nasal swab; CE, ISO13485]. https://www.bioteke.cn/5-In-1-Multiple-Respiratory-Multipathogen-Antigen-Test-Kit-pd791991098.html. Accessed.

[15] Nacov JA, Salmanton-García J, Grimm S, Stewart FA, Cremer LM, Rochel LM, et al. Self-testing for 5 respiratory viruses in adult VACCELERATE volunteers in Germany-a pilot study on multi-pathogen rapid antigen testing to monitor community-acquired acute respiratory infections. Front Public Health. 2025;13:1638280. 10.3389/fpubh.2025.1638280. PMC12440861 40969624

[16] Bosch W, Speiser LJ, Wi C-I, King KS, Natoli TL, Ihrke KD, et al. Incidence of respiratory syncytial virus in community-dwelling adults aged 18–64 years over 2 seasons, 2022–2024, in a North American community. Open Forum Infect Dis. 2022. 10.1093/ofid/ofae597. PMC11521338 39474445

[17] Yasuda I, Suzuki M, Maeda H, Terada M, Sando E, Ng CFS, et al. Respiratory virus detection in the upper respiratory tract of asymptomatic, community-dwelling older people. BMC Infect Dis. 2022;22(1):411. 10.1186/s12879-022-07355-w. PMC9047617 35484482

[18] Buda SDR, Biere B, Reiche J, Buchholz U, Tolksdorf K, Gvaladze T, Schilling J, Preuß U, Prahm K, Krupka S, Lehfeld AS, Haas W, ARE-Wochenbericht. KW 22/2024. 10.25646/12113

[19] Buda SDR, Biere B, Reiche J, Buchholz U, Tolksdorf K, Gvaladze T, Schilling J, Lehfeld AS, Cai W, Kerber R, Preuß HC, Prahm U, Krupka K, Haas S. W. ARE-Wochenbericht KW 50/2024. 10.25646/12957

[20] Garnett CT, Talekar G, Mahr JA, Huang W, Zhang Y, Ornelles DA, et al. Latent Species C Adenoviruses in Human Tonsil Tissues. J Virol. 2009;83(6):2417–28. 10.1128/jvi.02392-08. PMC2648257 19109384

[21] van Son JE, Kahn ECP, van der Bol JM, Barten DG, Blomaard LC, van Dam C, et al. Atypical presentation of COVID-19 in older patients is associated with frailty but not with adverse outcomes. Eur Geriatr Med. 2023;14(2):333–43. 10.1007/s41999-022-00736-z. PMC9902812 36749454

[22] Zheng W, Kämpfen F, Huang Z. Health-seeking and diagnosis delay and its associated factors: a case study on COVID-19 infections in Shaanxi Province, China. Sci Rep. 2021;11(1):17331. 10.1038/s41598-021-96888-2. PMC8405662 34462494

[23] Robert Koch-Institut. ARE-Dashboard und weitere Daten. https://www.rki.de/DE/Themen/Infektionskrankheiten/Akute-respiratorische-Erkrankungen/Dashboard_Daten.html. Accessed.

[24] Lynch JP 3rd, Kajon AE. Adenovirus: epidemiology, global spread of novel serotypes, and advances in treatment and prevention. Semin Respir Crit Care Med. 2016;37(4):586–602. 10.1055/s-0036-1584923. PMC7171713 27486739

[25] Zhang L, Guo Y, Wang X, Gai W, Liu L. Severe adenovirus pneumonia complicated by acute respiratory distress syndrome in immunocompetent patients: a case report and literature review. Front Med. 2025. 10.3389/fmed.2025.1524783. PMC11955468 40166061

[26] Chahal JS, Qi J, Flint SJ. The human adenovirus type 5 E1B 55 kDa protein obstructs inhibition of viral replication by type I interferon in normal human cells. PLoS Pathog. 2012;8(8):e1002853. 10.1371/journal.ppat.1002853. PMC3415460 22912576

[27] Price RHM, Graham C, Ramalingam S. Association between viral seasonality and meteorological factors. Sci Rep. 2019;9(1):929. 10.1038/s41598-018-37481-y. PMC6353886 30700747

[28] Shan S, Zhang W, Gao H, Huang P-Y, Du Z, Bai Y, et al. Global seasonal activities of respiratory syncytial virus before the coronavirus disease 2019 pandemic: a systematic review. Open Forum Infect Dis. 2019. 10.1093/ofid/ofae238. PMC11103620 38770210

[29] Loosen SH, Plendl W, Konrad M, Tanislav C, Luedde T, Roderburg C, et al. Prevalence of upper respiratory tract infections before, during, and after the COVID-19 pandemic in Germany: a cross-sectional study of 2 167 453 outpatients. J Prim Care Community Health. 2023;14:21501319231204436. 10.1177/21501319231204436. PMC10580722 37846103

[30] Badura B, Ducki A, Baumgardt J, Meyer M, Schröder H. Fehlzeiten-Report 2024: Bindung und Gesundheit - Fachkräfte gewinnen und halten. Springer Berlin Heidelberg; 2024.

[31] Statistisches Bundesamt. Krankenstand. (2026). https://www.destatis.de/DE/Themen/Arbeit/Arbeitsmarkt/Qualitaet-Arbeit/Dimension-2/krankenstand.html. Accessed January 20, 2026.

[32] Kuster SP, Böni J, Kouyos RD, Huber M, Schmutz S, Shah C, et al. Absenteeism and presenteeism in healthcare workers due to respiratory illness. Infect Control Hosp Epidemiol. 2021;42(3):268–73. 10.1017/ice.2020.444. 33239124

[33] Deutsches Ärzteblatt. Debatte um Fehltage: Merz übt Kritik, Unterstützung von der KBV. (2026). https://www.aerzteblatt.de/news/debatte-um-fehltage-merz-ubt-kritik-unterstutzung-von-der-kbv-c124be87-8a3d-4eb3-9407-f6715d1778d3. Accessed January 20, 2026.

[34] Ziebarth N. Karenztage und Absenkung der Lohnersatzrate – eine ökonomische Einordnung. 2025. pp. 675 – 85.

[35] Yang J, Rai KK, Seif M, Volkman HR, Ren J, Schmetz A, et al. COVID-19-related work absenteeism and associated lost productivity cost in Germany: a population-based study. J Occup Environ Med. 2024;66(6):514–22. 10.1097/jom.0000000000003093. 38489399

[36] Wanger S, Weber E. Sickness absence due to mandatory COVID-19 certificates in the workplace. BMC Public Health. 2023;23(1):1482. 10.1186/s12889-023-16415-y. PMC10401870 37537556

[37] Ahsan A, Chaudhry A, Shabbir, Qadeer MA, Rafiq A, Yaseen A et al. Comparison of rapid antigen test with RT-PCR for COVID-19 diagnosis: performance and limitation. 2024. 10.15275/rusomj.2024.0210

[38] Chartrand C, Tremblay N, Renaud C, Papenburg J. Diagnostic accuracy of rapid antigen detection tests for respiratory syncytial virus infection: systematic review and meta-analysis. J Clin Microbiol. 2015;53(12):3738–49. 10.1128/jcm.01816-15. PMC4652120 26354816

[39] Nacov JA, Salmanton-García J, Jakobs J, Anggitakirana M, Grimm S, Leckler J, et al. Self-testing for five respiratory viruses in adults ≥ 50 years of age in Germany for longitudinal monitoring of community-acquired acute respiratory infections. Clin Microbiol Infect. 2026. 10.1016/j.cmi.2026.03.005. 41856404

