# Supplementary material for: Point prevalence assessments of five respiratory viruses in adults ≥ 50 years of age in Germany by multi-pathogen rapid antigen testing on June 1, 2024, and December 15, 2024
Source: Infection. 2026 Aug 25;54(5):2725–38. doi: 10.1007/s15010-026-02917-5 (PMC13727950; doi:10.1007/s15010-026-02917-5)
Supplement: Supplementary file 1 [file 15010_2026_2917_MOESM1_ESM.docx]

*Research*

**Point prevalence assessments of five respiratory viruses in adults ≥50 years of age in Germany by multi-pathogen rapid antigen testing on June 1, 2024, and December 15, 2024**

**Authors**

Miranda Anggitakirana^1,2,3^*, Julia A. Nacov^1,2,3^*, Jon Salmanton-García^1,2,3^, Julia Jakobs^1,2,3^, Sarah Grimm^1,2,3^, Janina Leckler^1,2,3^, Katharina Köbe^1,2,3^, Louise M. Cremer^1,2,3^, Pauline Wipfler^1,2,3^, Jannik Stemler^1,2,3^, Heinz-Josef Schmitt^1,2,3^, Ullrich Bethe^1,2,3^, Oliver A. Cornely^1,2,3,4^

*These authors contributed equally to this work and share first authorship.

**Affiliations**

1) University of Cologne, Faculty of Medicine and University Hospital Cologne, Institute of Translational Research, Cologne Excellence Cluster on Cellular Stress Responses in Aging-Associated Diseases (CECAD), Cologne, Germany
2) University of Cologne, Faculty of Medicine, and University Hospital Cologne,

Department I of Internal Medicine, Excellence Center for Medical Mycology (ECMM), Cologne, Germany

3) German Centre for Infection Research (DZIF), Partner Site Bonn-Cologne, Cologne, Germany
4) University of Cologne, Faculty of Medicine and University Hospital Cologne, Clinical Trials Centre Cologne (ZKS Köln), Cologne, Germany

**Author ORCID ID**

| Miranda Anggitakirana | 0009-0007-3783-3419 |
| --- | --- |
| Julia A. Nacov | 0000-0002-5327-2937 |
| Jon Salmanton-García | 0000-0002-6766-8297 |
| Julia Jakobs | 0000-0002-4669-690X |
| Sarah Grimm | 0000-0001-7680-0344 |
| Louise M. Cremer | 0009-0002-1425-7352 |
| Pauline Wipfler | 0000-0003-0400-0748 |
| Jannik Stemler | 0000-0001-9152-2469 |
| Heinz-Josef Schmitt | 0000-0002-0378-5679 |
| Ullrich Bethe | 0000-0001-9979-1671 |
| Oliver A. Cornely | 0000-0001-9599-3137 |

**Key words**

Rapid antigen test, MAK5, viral respiratory tract infections, AdV, SARS-CoV-2, influenza

**Corresponding author**

Oliver A. Cornely, MD
Institute of Translational Research, Cologne Excellence Cluster on Cellular Stress Responses in Aging-Associated Diseases (CECAD)

University of Cologne, Faculty of Medicine, and University Hospital of Cologne

Herderstr. 52

50931 Cologne, Germany

Phone: +49 (0) 221 478 39391
Fax: +49 (0) 221 478 1421445
E-Mail: [oliver.cornely@uk.koeln.de](mailto:oliver.cornely@uk.koeln.de)

**Supplementary Material**

1 Supplementary Tables

Supplementary Table 1	4

Supplementary Table 2	5

Supplementary Table 3	6

Supplementary Table 4	7

Supplementary Table 5	8

Supplementary Table 6	11

Supplementary Table 7	12

Supplementary Table 8	15

2 Supplementary Figures

Supplementary Figure 1	16

Supplementary Figure 2	17

Supplementary Figure 3	18

Supplementary Figure 4	19

Supplementary Figure 5	20

Supplementary Figure 6	21

**Supplementary Table 1.** **Clinical performance of the Multipathogen Antigen Test Kit (MAK5)**

|  | Sensitivity  [95% CI] | Specificity  [95% CI] | Coincidence rate  [95% CI] | Kappa-value |
| --- | --- | --- | --- | --- |
| SARS-CoV-2 | 96.36  [90.95; 99.00] | 100  [99.19; 100.00] | 99.29  [98.20; 99.81] | 0.9771 |
| Influenza A virus | 94.96  [89.35; 98.13] | 100  [99.18; 100] | 98.95  [97.71; 99.61] | 0.9675 |
| Influenza B virus | 95.37  [89.53; 98.48] | 100  [99.18; 100] | 99.10  [97.91; 99.71] | 0.9707 |
| RSV A/B | 93.81  [87.65; 97.47] | 100  [99.19; 100] | 98.77  [97.47; 99.50] | 0.9604 |
| AdV | 94.29  [87.98; 97.87] | 100  [99.19; 100] | 98.93  [97.68; 99.61] | 0.9640 |

*Data provided by the manufacturer BioTeke Corporation (Wuxi) Co., Ltd., Wuxi, Jiangsu, China on 25 May, 2023. Evaluated with clinical samples by comparison with test results from the same samples using light cycler PCR (Roche) and Cobas Z480 Analyzer (Roche).CI Confidence Interval*

**Supplementary Table 2. Detections (including co-detections) at the point prevalence assessments on June 1, 2024, and December 15, 2024**

| **Test result** | **June 1, 2024**  **N [%]** | **December 15, 2024**  **N [%]** | **TOTAL N [%]** |
| --- | --- | --- | --- |
| Invalid test | 12 [0.4] | 9 [0.4] | 21 [0.4] |
| Negative test | 2536 [93.9] | 2271 [93.9] | 4807 [93.9] |
| SARS-CoV-2 | 40 [1.5] | 36 [1.5] | 76 [1.5] |
| Influenza A virus | 36 [1.3] | 40 [1.7] | 76 [1.5] |
| Influenza B virus | 7 [0.3] | 6 [0.2] | 13 [0.3] |
| RSV | 11 [0.4] | 12 [0.5] | 23 [0.4] |
| AdV | 59 [2.2] | 44 [1.8] | 103 [2.0] |
| **TOTAL** | 2701 [100.0] | 2418 [100.0] | 5119 [100.0] |

*AdV, adenovirus; RSV, respiratory syncytial virus; SARS-CoV-2, severe acute respiratory syndrome coronavirus 2*

**Supplementary Table 3. Co-detections at the point prevalence assessments on June 1, 2024, and December 15, 2024**

| **Test result** | **June 1, 2024**  **N [%]** | **December 15, 2024**  **N [%]** | **TOTAL N [%]** |
| --- | --- | --- | --- |
| AdV + Influenza A virus | 16 [55.2] | 10 [50.0] | 26 [53.1] |
| AdV + Influenza A- + Influenza B virus + RSV + SARS-CoV-2 | 4 [13.8] | 2 [10.0] | 6 [12.2] |
| AdV + Influenza A virus + RSV | 5 [17.2] | 5 [25.0] | 10 [20.4] |
| AdV + Influenza B virus + SARS-CoV-2 | 1 [3.4] | 0 [0.0] | 1 [2.0] |
| AdV + SARS-CoV-2 | 2 [6.9] | 0 [0.0] | 2 [4.1] |
| Influenza A- + Influenza B virus | 1 [3.4] | 0 [0.0] | 1 [2.0] |
| Influenza A- + Influenza B virus + RSV + SARS-CoV-2 | 0 [0.0] | 1 [5.0] | 1 [2.0] |
| Influenza B virus + SARS-CoV-2 | 0 [0.0] | 2 [10.0] | 2 [4.1] |
| **TOTAL** | 29 [59.2] | 20 [40.8] | 49 [100.0] |

*AdV, adenovirus; RSV, respiratory syncytial virus; SARS-CoV-2, severe acute respiratory syndrome coronavirus 2*

**Supplementary Table 4. Single pathogen only detections at the point prevalence assessments on June 1, 2024, and December 15, 2024**

| **Test result** | **June 1, 2024**  **N [%]** | **December 15, 2024**  **N [%]** | **TOTAL N [%]** |
| --- | --- | --- | --- |
| SARS-CoV-2 | 33 [42.9] | 31 [36.5] | 64 [39.5] |
| Influenza A virus | 10 [13.0] | 22 [25.9] | 32 [19.8] |
| Influenza B virus | 1 [1.3] | 1 [1.2] | 2 [1.2] |
| RSV | 2 [2.6] | 4 [4.7] | 6 [3.7] |
| AdV | 31 [40.3] | 27 [31.8] | 58 [35.8] |
| **TOTAL** | 77 [8.9] | 85 [9.9] | 162 [100.0] |

*AdV, adenovirus; RSV, respiratory syncytial virus; SARS-CoV-2, severe acute respiratory syndrome coronavirus 2*

**Supplementary Table 5. Cohort demographics and pre-existing conditions among participants by test result at point prevalence assessment on June 1, 2024**

|  | **Participants**  **with**  **invalid test**  **N [%]** | **Participants**  **with**  **negative test**  **N [%]** | **Participants**  **positive for**  **SARS-CoV-2**  **N [%]** | **Participants**  **positive for**  **influenza A virus**  **N [%]** | **Participants**  **positive for**  **influenza B virus**  **N [%]** | **Participants**  **positive for**  **RSV**  **N [%]** | **Participants**  **positive for**  **AdV**  **N [%]** | **TOTAL**  **N [%]** |
| --- | --- | --- | --- | --- | --- | --- | --- | --- |
| **TOTAL** | 12 [0.4%] | 2536 [93.9%] | 40 [1.5%] | 36 [1.3%] | 7 [0.3%] | 11 [0.4%] | 59 [2.2%] | 2654 [100] |
|  |  |  |  |  |  |  |  |  |
| **Gender** |  |  |  |  |  |  |  |  |
| Female | 7 [0.5] | 1480 [95.4] | 23 [1.5] | 26 [1.7] | 4 [0.3] | 8 [0.5] | 38 [2.4] | 1552 [58.5] |
| Male | 5 [0.5] | 1055 [95.8] | 17 [1.5] | 10 [0.9] | 3 [0.3] | 3 [0.3] | 21 [1.9] | 1101 [41.5] |
| Non-binary* | 0 [0.0] | 1 [100.0] | 0 [0.0] | 0 [0.0] | 0 [0.0] | 0 [0.0] | 0 [0.0] | 1 [0.0] |
|  |  |  |  |  |  |  |  |  |
| **Age (years)** |  |  |  |  |  |  |  |  |
| Median [min-max] | 65 [50-87] | 59 [50-91] | 60 [50-76] | 58 [50-76] | 69 [60-76] | 58 [54-76] | 57 [50-76] | 59 [50-91] |
|  |  |  |  |  |  |  |  |  |
| **Age groups** |  |  |  |  |  |  |  |  |
| 50 to 60 years | 3 [0.2] | 1315 [95.4] | 20 [1.5] | 21 [1.5] | 0 [0.0] | 6 [0.4] | 35 [2.5] | 1378 [51.9] |
| 60 to 70 years | 6 [0.6] | 899 [95.5] | 15 [1.6] | 11 [1.2] | 4 [0.4] | 3 [0.3] | 18 [1.9] | 941 [35.5] |
| 70 to 80 years | 1 [0.3] | 291 [96.4] | 5 [1.7] | 4 [1.3] | 3 [1.0] | 2 [0.7] | 6 [2.0] | 302 [11.4] |
| 80 years and above | 2 [6.1] | 31 [93.9] | 0 [0.0] | 0 [0.0] | 0 [0.0] | 0 [0.0] | 0 [0.0] | 33 [1.2] |
|  |  |  |  |  |  |  |  |  |
| **Pre-existing conditions** |  |  |  |  |  |  |  |  |
| Hypertension | 3 [0.4] | 682 [95.5] | 13 [1.8] | 7 [1.0] | 2 [0.3] | 4 [0.6] | 14 [2.0] | 714 [26.9] |
| Coronary heart disease | 1 [1.0] | 94 [96.9] | 1 [1.0] | 0 [0.0] | 0 [0.0] | 0 [0.0] | 1 [1.0] | 97 [3.7] |
| Heart failure | 0 [0.0] | 35 [92.1] | 1 [2.6] | 0 [0.0] | 1 [2.6] | 0 [0.0] | 3 [7.9] | 38 [1.4] |
| Chronic lung disease | 2 [0.7] | 275 [94.2] | 6 [2.1] | 6 [2.1] | 1 [0.3] | 1 [0.3] | 10 [3.4] | 292 [11.0] |
| Chronic hepatitis B or C | 0 [0.0] | 0 [0.0] | 0 [0.0] | 0 [0.0] | 0 [0.0] | 0 [0.0] | 0 [0.0] | 0 [0.0] |
| Chronic non-infectious liver disease | 0 [0.0] | 16 [94.1] | 0 [0.0] | 1 [5.9] | 0 [0.0] | 0 [0.0] | 1 [5.9] | 17 [0.6] |
| Chronic kidney disease | 0 [0.0] | 34 [97.1] | 0 [0.0] | 0 [0.0] | 0 [0.0] | 0 [0.0] | 1 [2.9] | 35 [1.3] |
| ≥20 kg overweight | 2 [0.6] | 294 [95.1] | 7 [2.3] | 4 [1.3] | 4 [1.3] | 3 [1.0] | 5 [1.6] | 309 [11.6] |
| Diabetes mellitus | 1 [0.9] | 109 [95.6] | 2 [1.8] | 2 [1.8] | 1 [0.9] | 2 [1.8] | 2 [1.8] | 114 [4.3] |
| HIV | 0 [0.0] | 21 [95.5] | 0 [0.0] | 0 [0.0] | 0 [0.0] | 0 [0.0] | 1 [4.5] | 22 [0.8] |
| Active cancer (current therapy or within last 2 yrs) | 0 [0.0] | 55 [93.2] | 1 [1.7] | 2 [3.4] | 1 [1.7] | 1 [1.7] | 4 [6.8] | 59 [2.2] |
| Epilepsy | 0 [0.0] | 12 [92.3] | 1 [7.7] | 0 [0.0] | 1 [7.7] | 0 [0.0] | 1 [7.7] | 13 [0.5] |
| Chronic GIT disease | 0 [0.0] | 29 [100.0] | 0 [0.0] | 0 [0.0] | 0 [0.0] | 0 [0.0] | 0 [0.0] | 29 [1.1] |
| Severe musculoskeletal condition | 0 [0.0] | 61 [100.0] | 0 [0.0] | 0 [0.0] | 0 [0.0] | 0 [0.0] | 0 [0.0] | 61 [2.3] |
| Psychiatric condition | 1 [0.7] | 130 [94.9] | 2 [1.5] | 2 [1.5] | 1 [0.7] | 0 [0.0] | 3 [2.2] | 137 [5.2] |
| History of stroke | 0 [0.0] | 18 [85.7] | 1 [4.8] | 0 [0.0] | 0 [0.0] | 0 [0.0] | 2 [9.5] | 21 [0.8] |
| Other | 6 [0.8] | 749 [95.2] | 12 [1.5] | 15 [1.9] | 1 [0.1] | 3 [0.4] | 19 [2.4] | 787 [29.7] |
|  |  |  |  |  |  |  |  |  |
| **Previously confirmed infections** |  |  |  |  |  |  |  |  |
| Influenza | 0 [0.0] | 234 [96.3] | 4 [1.6] | 3 [1.2] | 0 [0.0] | 0 [0.0] | 4 [1.6] | 243 [9.2] |
| COVID-19 | 10 [0.5] | 1960 [95.1] | 35 [1.7] | 32 [1.6] | 4 [0.2] | 10 [0.5] | 50 [2.4] | 2061 [77.7] |
| RSV infection | 0 [0.0] | 2 [100.0] | 0 [0.0] | 0 [0.0] | 0 [0.0] | 0 [0.0] | 0 [0.0] | 2 [0.1] |
| AdV infection | 0 [0.0] | 10 [90.9] | 0 [0.0] | 0 [0.0] | 0 [0.0] | 0 [0.0] | 1 [9.1] | 11 [0.4] |
|  |  |  |  |  |  |  |  |  |
| **Seasonal influenza vaccination** |  |  |  |  |  |  |  |  |
| Influenza vaccination received in 2019 | 0 [0.0] | 25 [100.0] | 0 [0.0] | 0 [0.0] | 0 [0.0] | 0 [0.0] | 0 [0.0] | 25 [0.9] |
| Influenza vaccination received in 2020 | 0 [0.0] | 25 [92.6] | 1 [3.7] | 0 [0.0] | 0 [0.0] | 0 [0.0] | 1 [3.7] | 27 [1.0] |
| Influenza vaccination received in 2021 | 0 [0.0] | 61 [100.0] | 0 [0.0] | 0 [0.0] | 0 [0.0] | 0 [0.0] | 0 [0.0] | 61 [2.3] |
| Influenza vaccination received in 2022 | 1 [0.6] | 147 [94.8] | 3 [1.9] | 1 [0.6] | 0 [0.0] | 0 [0.0] | 4 [2.6] | 155 [5.8] |
| Influenza vaccination received in 2023 | 7 [0.7] | 1015 [94.5] | 24 [2.2] | 15 [1.4] | 4 [0.4] | 6 [0.6] | 32 [3.0] | 1074 [40.5] |
| Influenza vaccination received in 2024 | 1 [0.1] | 727 [96.3] | 7 [0.9] | 11 [1.5] | 3 [0.4] | 4 [0.5] | 14 [1.9] | 755 [28.4] |
|  |  |  |  |  |  |  |  |  |
| **COVID-19 vaccination** |  |  |  |  |  |  |  |  |
| Total number of COVID-19 vaccinations received | 12 [0.5] | 2490 [95.5] | 40 [1.5] | 35 [1.3] | 7 [0.3] | 11 [0.4] | 57 [2.2] | 2606 [98.2] |
| Mean ± SD | 4 [1-6] | 4 [0-10] | 4 [2-6] | 4 [2-6] | 5 [3-6] | 4 [3-6] | 4 [3-7] | 4 [0-10] |
| Median [min-max] |  |  |  |  |  |  |  |  |
| Last COVID-19 vaccination received in 2020 | 0 [0.0] | 5 [100.0] | 0 [0.0] | 0 [0.0] | 0 [0.0] | 0 [0.0] | 0 [0.0] | 5 [0.2] |
| Last COVID-19 vaccination received in 2021 | 2 [0.3] | 579 [96.2] | 6 [1.0] | 10 [1.7] | 1 [0.2] | 1 [0.2] | 12 [2.0] | 602 [22.7] |
| Last COVID-19 vaccination received in 2022 | 4 [0.5] | 748 [94.8] | 14 [1.8] | 11 [1.4] | 1 [0.1] | 3 [0.4] | 21 [2.7] | 789 [29.7] |
| Last COVID-19 vaccination received in 2023 | 6 [0.8] | 747 [94.7] | 18 [2.3] | 11 [1.4] | 5 [0.6] | 6 [0.8] | 17 [2.2] | 789 [29.7] |
| Last COVID-19 vaccination received in 2024 | 0 [0.0] | 387 [97.7] | 2 [0.5] | 2 [0.5] | 0 [0.0] | 1 [0.3] | 7 [1.8] | 396 [14.9] |
| Last COVID-19 vaccination received = BioNTech | 11 [0.5] | 2022 [95.6] | 30 [1.4] | 27 [1.3] | 6 [0.3] | 9 [0.4] | 44 [2.1] | 2115 [79.7] |
| Last COVID-19 vaccination received = Moderna | 0 [0.0] | 399 [95.0] | 9 [2.1] | 8 [1.9] | 1 [0.2] | 2 [0.5] | 13 [3.1] | 420 [15.8] |
| Last COVID-19 vaccination received = Other | 1 [1.4] | 69 [97.2] | 1 [1.4] | 0 [0.0] | 0 [0.0] | 0 [0.0] | 0 [0.0] | 71 [2.7] |
|  |  |  |  |  |  |  |  |  |
| **RSV vaccination** |  |  |  |  |  |  |  |  |
| Received RSV vaccination in 2023 | 0 [0.0] | 14 [93.3] | 0 [0.0] | 0 [0.0] | 0 [0.0] | 0 [0.0] | 1 [6.7] | 15 [0.6] |
| Received RSV vaccination in 2024 | 0 [0.0] | 26 [96.3] | 0 [0.0] | 0 [0.0] | 0 [0.0] | 0 [0.0] | 1 [3.7] | 27 [1.0] |

*AdV, adenovirus; COPD, chronic obstructive pulmonary disease; COVID-19, coronavirus disease 2019; GIT, gastrointestinal; HIV, human immunodeficiency virus; INF, infection; RSV, respiratory syncytial virus; SARS-CoV-2, severe acute respiratory syndrome coronavirus 2; yrs., years.*

*Corresponds to legal term “divers” in German language.

**Supplementary Table 6. Geographical distribution of participants at point prevalence assessment on June 1, 2024**

| **Place of residence** | **Participants**  **with**  **invalid test**  **N [%]** | **Participants**  **with**  **negative test**  **N [%]** | **Participants**  **positive for**  **SARS-CoV-2**  **N [%]** | **Participants**  **positive for**  **influenza A virus**  **N [%]** | **Participants**  **positive for**  **influenza B virus**  **N [%]** | **Participants**  **positive for**  **RSV**  **N [%]** | **Participants**  **positive for**  **AdV**  **N [%]** | **TOTAL**  **N [%]** |
| --- | --- | --- | --- | --- | --- | --- | --- | --- |
| Baden-Wuerttemberg | [0.0] | 227 [96.2] | 3 [1.3] | 6 [2.5] | 1 [0.4] | 3 [1.3] | 5 [2.1] | 236 [8.9] |
| Bavaria | 0 [0.0] | 217 [96.0] | 1 [0.4] | 4 [1.8] | 1 [0.4] | 3 [1.3] | 7 [3.1] | 226 [8.5] |
| Berlin | 0 [0.0] | 119 [96.7] | 3 [2.4] | 0 [0.0] | 0 [0.0] | 0 [0.0] | 1 [0.8] | 123 [4.6] |
| Brandenburg | 1 [2.0] | 45 [91.8] | 1 [2.0] | 1 [2.0] | 1 [2.0] | 0 [0.0] | 2 [4.1] | 49 [1.8] |
| Bremen | 0 [0.0] | 18 [94.7] | 1 [5.3] | 0 [0.0] | 0 [0.0] | 0 [0.0] | 0 [0.0] | 19 [0.7] |
| Hamburg | 0 [0.0] | 44 [95.7] | 2 [4.3] | 0 [0.0] | 0 [0.0] | 0 [0.0] | 0 [0.0] | 46 [1.7] |
| Hesse | 0 [0.0] | 212 [96.4] | 3 [1.4] | 2 [0.9] | 0 [0.0] | 1 [0.5] | 4 [1.8] | 220 [8.3] |
| Lower Saxony | 0 [0.0] | 163 [97.6] | 1 [0.6] | 1 [0.6] | 1 [0.6] | 0 [0.0] | 1 [0.6] | 167 [6.3] |
| Mecklenburg-Western Pomerania | 0 [0.0] | 18 [94.7] | 1 [5.3] | 1 [5.3] | 1 [5.3] | 1 [5.3] | 1 [5.3] | 19 [0.7] |
| North Rhine-Westphalia | 10 [0.8] | 1182 [94.9] | 23 [1.8] | 16 [1.3] | 2 [0.2] | 2 [0.2] | 29 [2.3] | 1246 [46.9] |
| Rhineland-Palatinate | 1 [0.9] | 107 [92.2] | 1 [0.9] | 4 [3.4] | 0 [0.0] | 1 [0.9] | 7 [6.0] | 116 [4.4] |
| Saarland | 0 [0.0] | 15 [100.0] | 0 [0.0] | 0 [0.0] | 0 [0.0] | 0 [0.0] | 0 [0.0] | 15 [0.6] |
| Saxony | 0 [0.0] | 49 [100.0] | 0 [0.0] | 0 [0.0] | 0 [0.0] | 0 [0.0] | 0 [0.0] | 49 [1.8] |
| Saxony-Anhalt | 0 [0.0] | 28 [93.3] | 0 [0.0] | 1 [3.3] | 0 [0.0] | 0 [0.0] | 1 [3.3] | 30 [1.1] |
| Schleswig-Holstein | 0 [0.0] | 64 [100.0] | 0 [0.0] | 0 [0.0] | 0 [0.0] | 0 [0.0] | 0 [0.0] | 64 [2.4] |
| Thuringia | 0 [0.0] | 28 [96.6] | 0 [0.0] | 0 [0.0] | 0 [0.0] | 0 [0.0] | 1 [3.4] | 29 [1.1] |
| **TOTAL** | **12 [0.5]** | **2536 [95.6]** | **40 [1.5]** | **36 [1.4]** | **7 [0.3]** | **11 [0.4]** | **59 [2.2]** | **2654 [100.0]** |

*AdV, adenovirus; RSV, respiratory syncytial virus; SARS-CoV-2, severe acute respiratory syndrome coronavirus 2.*

*Please note: A single participants may account for multiple detections*

**Supplementary Table 7. Cohort demographics and pre-existing conditions among participants by test result at point prevalence assessment on December 15, 2024**

|  | **Participants**  **with**  **invalid test**  **N [%]** | **Participants**  **with**  **negative test**  **N [%]** | **Participants**  **positive for**  **SARS-CoV-2**  **N [%]** | **Participants**  **positive for**  **influenza A virus**  **N [%]** | **Participants**  **positive for**  **influenza B virus**  **N [%]** | **Participants**  **positive for**  **RSV**  **N [%]** | **Participants**  **positive for**  **AdV**  **N [%]** | **TOTAL**  **N [%]** |
| --- | --- | --- | --- | --- | --- | --- | --- | --- |
| **TOTAL** | 9 [0.4%] | 2271 [93.9%] | 36 [1.5%] | 40 [1.7%] | 6 [0.2%] | 12 [0.5%] | 44 [1.8%] | 2385 [100] |
|  |  |  |  |  |  |  |  |  |
| **Gender** |  |  |  |  |  |  |  |  |
| Female | 5 [0.4] | 1297 [94.4] | 23 [1.7] | 32 [2.3] | 5 [0.4] | 11 [0.8] | 32 [2.3] | 1374 [57.6] |
| Male | 4 [0.4] | 974 [96.4] | 13 [1.3] | 8 [0.8] | 1 [0.1] | 1 [0.1] | 11 [1.1] | 1010 [42.3] |
| Non-binary* | 0 [0.0] | 0 [0.0] | 0 [0.0] | 0 [0.0] | 0 [0.0] | 0 [0.0] | 1 [100.0] | 1 [0.0] |
|  |  |  |  |  |  |  |  |  |
| **Age (years)** |  |  |  |  |  |  |  |  |
| Median [min-max] | 59 [52-80] | 60 [50-91] | 59 [50-81] | 57 [50-78] | 57 [51-67] | 58 [53-70] | 56 [50-73] | 60 [50-91] |
|  |  |  |  |  |  |  |  |  |
| **Age groups** |  |  |  |  |  |  |  |  |
| 50 to 60 years | 5 [0.4] | 1112 [94.2] | 19 [1.6] | 26 [2.2] | 3 [0.3] | 7 [0.6] | 34 [2.9] | 1181 [49.5] |
| 60 to 70 years | 2 [0.2] | 855 [96.3] | 15 [1.7] | 8 [0.9] | 3 [0.3] | 4 [0.5] | 8 [0.9] | 888 [37.2] |
| 70 to 80 years | 1 [0.3] | 280 [96.6] | 1 [0.3] | 6 [2.1] | 0 [0.0] | 1 [0.3] | 2 [0.7] | 290 [12.2] |
| 80 years and above | 1 [3.8] | 24 [92.3] | 1 [3.8] | 0 [0.0] | 0 [0.0] | 0 [0.0] | 0 [0.0] | 26 [1.1] |
|  |  |  |  |  |  |  |  |  |
| **Pre-existing conditions** |  |  |  |  |  |  |  |  |
| Hypertension | 2 [0.3] | 604 [95.9] | 12 [1.9] | 10 [1.6] | 1 [0.2] | 1 [0.2] | 5 [0.8] | 630 [26.4] |
| Coronary heart disease | 0 [0.0] | 84 [98.8] | 0 [0.0] | 0 [0.0] | 0 [0.0] | 0 [0.0] | 1 [1.2] | 85 [3.6] |
| Heart failure | 0 [0.0] | 37 [94.9] | 0 [0.0] | 1 [2.6] | 0 [0.0] | 1 [2.6] | 0 [0.0] | 39 [1.6] |
| Chronic lung disease | 2 [0.8] | 236 [92.9] | 7 [2.8] | 9 [3.5] | 3 [1.2] | 3 [1.2] | 6 [2.4] | 254 [10.6] |
| Chronic hepatitis B or C | 0 [0.0] | 0 [0.0] | 0 [0.0] | 0 [0.0] | 0 [0.0] | 0 [0.0] | 0 [0.0] | 0 [0.0] |
| Chronic non-infectious liver disease | 0 [0.0] | 12 [85.7] | 0 [0.0] | 2 [14.3] | 0 [0.0] | 0 [0.0] | 1 [7.1] | 14 [0.6] |
| Chronic kidney disease | 0 [0.0] | 29 [90.6] | 0 [0.0] | 3 [9.4] | 0 [0.0] | 0 [0.0] | 1 [3.1] | 32 [1.3] |
| ≥20 kg overweight | 0 [0.0] | 235 [94.4] | 7 [2.8] | 3 [1.2] | 1 [0.4] | 1 [0.4] | 6 [2.4] | 249 [10.4] |
| Diabetes mellitus | 0 [0.0] | 87 [92.6] | 3 [3.2] | 3 [3.2] | 1 [1.1] | 1 [1.1] | 3 [3.2] | 94 [3.9] |
| HIV | 0 [0.0] | 22 [100.0] | 0 [0.0] | 0 [0.0] | 0 [0.0] | 0 [0.0] | 0 [0.0] | 22 [0.9] |
| Active cancer (current therapy or within last 2 yrs) | 1 [1.9] | 49 [92.5] | 0 [0.0] | 3 [5.7] | 0 [0.0] | 1 [1.9] | 2 [3.8] | 53 [2.2] |
| Epilepsy | 0 [0.0] | 14 [100.0] | 0 [0.0] | 0 [0.0] | 0 [0.0] | 0 [0.0] | 0 [0.0] | 14 [0.6] |
| Chronic GIT disease | 0 [0.0] | 22 [100.0] | 0 [0.0] | 0 [0.0] | 0 [0.0] | 0 [0.0] | 0 [0.0] | 22 [0.9] |
| Severe musculoskeletal condition | 0 [0.0] | 37 [88.1] | 4 [9.5] | 0 [0.0] | 0 [0.0] | 1 [2.4] | 0 [0.0] | 42 [1.8] |
| Psychiatric condition | 0 [0.0] | 99 [90.0] | 5 [4.5] | 3 [2.7] | 1 [0.9] | 2 [1.8] | 6 [5.5] | 110 [4.6] |
| History of stroke | 0 [0.0] | 21 [95.5] | 1 [4.5] | 0 [0.0] | 0 [0.0] | 0 [0.0] | 0 [0.0] | 22 [0.9] |
| Other | 5 [0.7] | 675 [94.5] | 13 [1.8] | 12 [1.7] | 3 [0.4] | 7 [1.0] | 17 [2.4] | 714 [29.9] |
|  |  |  |  |  |  |  |  |  |
| **Previously confirmed infections** |  |  |  |  |  |  |  |  |
| Influenza | 2 [1.0] | 200 [97.1] | 3 [1.5] | 1 [0.5] | 0 [0.0] | 0 [0.0] | 1 [0.5] | 206 [8.6] |
| COVID-19 | 7 [0.4] | 1763 [94.9] | 32 [1.7] | 32 [1.7] | 6 [0.3] | 10 [0.5] | 35 [1.9] | 1857 [77.9] |
| RSV infection | 0 [0.0] | 2 [100.0] | 0 [0.0] | 0 [0.0] | 0 [0.0] | 0 [0.0] | 0 [0.0] | 2 [0.1] |
| AdV infection | 0 [0.0] | 8 [100.0] | 0 [0.0] | 0 [0.0] | 0 [0.0] | 0 [0.0] | 0 [0.0] | 8 [0.3] |
|  |  |  |  |  |  |  |  |  |
| **Seasonal influenza vaccination** |  |  |  |  |  |  |  |  |
| Influenza vaccination received in 2019 | 0 [0.0] | 22 [100.0] | 0 [0.0] | 0 [0.0] | 0 [0.0] | 0 [0.0] | 0 [0.0] | 22 [0.9] |
| Influenza vaccination received in 2020 | 1 [3.8] | 24 [92.3] | 0 [0.0] | 1 [3.8] | 0 [0.0] | 0 [0.0] | 0 [0.0] | 26 [1.1] |
| Influenza vaccination received in 2021 | 0 [0.0] | 51 [94.4] | 2 [3.7] | 0 [0.0] | 0 [0.0] | 0 [0.0] | 1 [1.9] | 54 [2.3] |
| Influenza vaccination received in 2022 | 0 [0.0] | 126 [95.5] | 1 [0.8] | 1 [0.8] | 0 [0.0] | 2 [1.5] | 4 [3.0] | 132 [5.5] |
| Influenza vaccination received in 2023 | 7 [0.7] | 913 [94.6] | 15 [1.6] | 21 [2.2] | 3 [0.3] | 4 [0.4] | 18 [1.9] | 965 [40.5] |
| Influenza vaccination received in 2024 | 1 [0.1] | 683 [95.4] | 8 [1.1] | 13 [1.8] | 2 [0.3] | 2 [0.3] | 14 [2.0] | 716 [30.0] |
|  |  |  |  |  |  |  |  |  |
| **COVID-19 vaccination** |  |  |  |  |  |  |  |  |
| Total number of COVID-19 vaccinations received | 9 [0.4] | 2236 [95.2] | 36 [1.5] | 40 [1.7] | 6 [0.3] | 12 [0.5] | 43 [1.8] | 2349 [98.5] |
| Mean ± SD |  |  |  |  |  |  |  |  |
| Median [min-max] | 5 [3-5] | 4 [0-10] | 4 [3-7] | 4 [3-7] | 5 [3-5] | 3 [3-5] | 4 [3-7] | 4 [0-10] |
| Last COVID-19 vaccination received in 2020 | 0 [0.0] | 3 [100.0] | 0 [0.0] | 0 [0.0] | 0 [0.0] | 0 [0.0] | 0 [0.0] | 3 [0.1] |
| Last COVID-19 vaccination received in 2021 | 2 [0.4] | 516 [95.0] | 9 [1.7] | 9 [1.7] | 1 [0.2] | 5 [0.9] | 14 [2.6] | 543 [22.8] |
| Last COVID-19 vaccination received in 2022 | 2 [0.3] | 650 [95.2] | 10 [1.5] | 8 [1.2] | 1 [0.1] | 3 [0.4] | 14 [2.0] | 683 [28.6] |
| Last COVID-19 vaccination received in 2023 | 5 [0.7] | 672 [95.0] | 9 [1.3] | 14 [2.0] | 2 [0.3] | 2 [0.3] | 10 [1.4] | 707 [29.6] |
| Last COVID-19 vaccination received in 2024 | 0 [0.0] | 383 [96.2] | 7 [1.8] | 8 [2.0] | 2 [0.5] | 2 [0.5] | 4 [1.0] | 398 [16.7] |
| Last COVID-19 vaccination received = BioNTech | 8 [0.4] | 1836 [95.4] | 27 [1.4] | 30 [1.6] | 4 [0.2] | 6 [0.3] | 29 [1.5] | 1924 [80.7] |
| Last COVID-19 vaccination received = Moderna | 1 [0.3] | 341 [93.4] | 9 [2.5] | 9 [2.5] | 2 [0.5] | 6 [1.6] | 14 [3.8] | 365 [15.3] |
| Last COVID-19 vaccination received = Other | 0 [0.0] | 59 [98.3] | 0 [0.0] | 1 [1.7] | 0 [0.0] | 0 [0.0] | 0 [0.0] | 60 [2.5] |
|  |  |  |  |  |  |  |  |  |
| **RSV vaccination** |  |  |  |  |  |  |  |  |
| Received RSV vaccination in 2023 | 0 [0.0] | 14 [100.0] | 0 [0.0] | 0 [0.0] | 0 [0.0] | 0 [0.0] | 0 [0.0] | 14 [0.6] |
| Received RSV vaccination in 2024 | 0 [0.0] | 23 [88.5] | 0 [0.0] | 1 [3.8] | 0 [0.0] | 0 [0.0] | 2 [7.7] | 26 [1.1] |

*AdV, adenovirus; COPD, chronic obstructive pulmonary disease; COVID-19, coronavirus disease 2019; GIT, gastrointestinal; HIV, human immunodeficiency virus; INF, infection; RSV, respiratory syncytial virus; SARS-CoV-2, severe acute respiratory syndrome coronavirus 2; yrs., years.*

*Corresponds to legal term “divers” in German language

**Supplementary Table 8. Geographical distribution of participants at point prevalence assessment on December 15, 2024**

| **Place of residence** | **Participants**  **with**  **invalid test**  **N [%]** | **Participants**  **with**  **negative test**  **N [%]** | **Participants**  **positive for**  **SARS-CoV-2**  **N [%]** | **Participants**  **positive for**  **influenza A virus**  **N [%]** | **Participants**  **positive for**  **influenza B virus**  **N [%]** | **Participants**  **positive for**  **RSV**  **N [%]** | **Participants**  **positive for**  **AdV**  **N [%]** | **TOTAL**  **N [%]** |
| --- | --- | --- | --- | --- | --- | --- | --- | --- |
| Baden-Wuerttemberg | 0 [0.0] | 227 [98.3] | 0 [0.0] | 2 [0.9] | 0 [0.0] | 2 [0.9] | 4 [1.7] | 231 [9.7] |
| Bavaria | 1 [0.5] | 187 [94.9] | 4 [2.0] | 1 [0.5] | 1 [0.5] | 2 [1.0] | 4 [2.0] | 197 [8.3] |
| Berlin | 2 [1.9] | 97 [90.7] | 3 [2.8] | 3 [2.8] | 0 [0.0] | 0 [0.0] | 4 [3.7] | 107 [4.5] |
| Brandenburg | 0 [0.0] | 49 [96.1] | 0 [0.0] | 2 [3.9] | 0 [0.0] | 0 [0.0] | 1 [2.0] | 51 [2.1] |
| Bremen | 0 [0.0] | 17 [94.4] | 1 [5.6] | 0 [0.0] | 0 [0.0] | 0 [0.0] | 0 [0.0] | 18 [0.8] |
| Hamburg | 0 [0.0] | 40 [93.0] | 1 [2.3] | 1 [2.3] | 0 [0.0] | 0 [0.0] | 1 [2.3] | 43 [1.8] |
| Hesse | 0 [0.0] | 181 [95.8] | 1 [0.5] | 3 [1.6] | 1 [0.5] | 2 [1.1] | 5 [2.6] | 189 [7.9] |
| Lower Saxony | 0 [0.0] | 144 [96.6] | 2 [1.3] | 2 [1.3] | 0 [0.0] | 0 [0.0] | 3 [2.0] | 149 [6.2] |
| Mecklenburg-Western Pomerania | 0 [0.0] | 17 [94.4] | 1 [5.6] | 1 [5.6] | 1 [5.6] | 1 [5.6] | 0 [0.0] | 18 [0.8] |
| North Rhine-Westphalia | 2 [0.2] | 1066 [95.3] | 20 [1.8] | 19 [1.7] | 3 [0.3] | 5 [0.4] | 17 [1.5] | 1119 [46.9] |
| Rhineland-Palatinate | 2 [1.8] | 104 [95.4] | 0 [0.0] | 3 [2.8] | 0 [0.0] | 0 [0.0] | 0 [0.0] | 109 [4.6] |
| Saarland | 0 [0.0] | 13 [92.9] | 0 [0.0] | 1 [7.1] | 0 [0.0] | 0 [0.0] | 0 [0.0] | 14 [0.6] |
| Saxony | 1 [2.1] | 42 [89.4] | 2 [4.3] | 1 [2.1] | 0 [0.0] | 0 [0.0] | 1 [2.1] | 47 [2.0] |
| Saxony-Anhalt | 0 [0.0] | 25 [96.2] | 0 [0.0] | 0 [0.0] | 0 [0.0] | 0 [0.0] | 1 [3.8] | 26 [1.1] |
| Schleswig-Holstein | 0 [0.0] | 43 [95.6] | 1 [2.2] | 0 [0.0] | 0 [0.0] | 0 [0.0] | 1 [2.2] | 45 [1.9] |
| Thuringia | 1 [4.5] | 19 [86.4] | 0 [0.0] | 1 [4.5] | 0 [0.0] | 0 [0.0] | 2 [9.1] | 22 [0.9] |
| **TOTAL** | **9 [0.4]** | **2271 [95.2]** | **36 [1.5]** | **40 [1.7]** | **6 [0.3]** | **12 [0.5]** | **44 [1.8]** | **2385 [100.0]** |

*AdV, adenovirus; RSV, respiratory syncytial virus; SARS-CoV-2, severe acute respiratory syndrome coronavirus 2
Please note: A single participants may be accounted for multiple time.*

**Supplementary Fig 1. Outline MAK5-50+ study**

*pp, point prevalence; T, time unit [month]*

**Supplementary Fig 2. User Instruction Multiple Respiratory Multipathogen Antigen Test Kit [15]**

*Image* *Source: BioTeke. 5 In 1 Multiple Respiratory Multipathogen Antigen Test Kit [SARS-CoV-2, Influenza A/B, RSV, ADV; anterior nasal swab; CE, ISO13485].* [*https://www.bioteke.cn/5-In-1-Multiple-Respiratory-Multipathogen-Antigen-Test-Kit-pd791991098.html*](https://www.bioteke.cn/5-In-1-Multiple-Respiratory-Multipathogen-Antigen-Test-Kit-pd791991098.html)*.*

**Supplementary Fig 3. Symptom Diary in German Language**

**Supplementary Fig 4. Geographical distribution of MAK5 test results on June 1, 2024**AdV, adenovirus; RSV, respiratory syncytial virus; SARS-CoV-2, severe acute respiratory syndrome coronavirus 2

**Supplementary Fig 5. Geographical distribution of MAK5 test results on December 15, 2024**

AdV, adenovirus; RSV, respiratory syncytial virus; SARS-CoV-2, severe acute respiratory syndrome coronavirus

**Supplementary Fig 6. Venn Diagram of Participant Participation in the Two Point-Prevalence Assessments**
